# Genome sequencing of *Sporisorium scitamineum* provides insights into the pathogenic mechanisms of sugarcane smut

**DOI:** 10.1186/1471-2164-15-996

**Published:** 2014-11-19

**Authors:** Youxiong Que, Liping Xu, Qibin Wu, Yongfeng Liu, Hui Ling, Yanhong Liu, Yuye Zhang, Jinlong Guo, Yachun Su, Jiebo Chen, Shanshan Wang, Chengguang Zhang

**Affiliations:** Key Laboratory of Sugarcane Biology and Genetic Breeding, Ministry of Agriculture, Fujian Agriculture and Forestry University, Fuzhou, 350002 China; BGI-Shenzhen, Main Building 11/F, Beishan Industrial Zone, Yantian District, Shenzhen, 518083 China; College of Life Science, Fujian Agriculture and Forestry University, Fuzhou, 350002 China

**Keywords:** *Sporisorium scitamineum*, Sugarcane smut, Pathogenic mechanisms, G-protein coupled receptors, Carbohydrate degrading enzymes, Biotrophic properties, Candidates for secreted effector proteins, Secondary metabolic pathways

## Abstract

**Background:**

Sugarcane smut can cause losses in cane yield and sugar content that range from 30% to total crop failure. Losses tend to increase with the passage of years. *Sporisorium scitamineum* is the fungus that causes sugarcane smut. This fungus has the potential to infect all sugarcane species unless a species is resistant to biotrophic fungal pathogens. However, it remains unclear how the fungus breaks through the cell walls of sugarcane and causes the formation of black or gray whip-like structures on the sugarcane plants.

**Results:**

Here, we report the first high-quality genome sequence of *S. scitamineum* assembled *de novo* with a contig N_50_ of 41 kb, a scaffold N_50_ of 884 kb and genome size 19.8 Mb, containing an estimated 6,636 genes. This phytopathogen can utilize a wide range of carbon and nitrogen sources. A reduced set of genes encoding plant cell wall hydrolytic enzymes leads to its biotrophic lifestyle, in which damage to the host should be minimized. As a bipolar mating fungus, *a* and *b* loci are linked and the mating-type locus segregates as a single locus. The *S. scitamineum* genome has only 6 G protein-coupled receptors (GPCRs) grouped into five classes, which are responsible for transducing extracellular signals into intracellular responses, however, the genome is without any PTH11-like GPCR. There are 192 virulence associated genes in the genome of *S. scitamineum*, among which 31 expressed in all the stages, which mainly encode for energy metabolism and redox of short-chain compound related enzymes. Sixty-eight candidates for secreted effector proteins (CSEPs) were found in the genome of *S. scitamineum*, and 32 of them expressed in the different stages of sugarcane infection, which are probably involved in infection and/or triggering defense responses. There are two non-ribosomal peptide synthetase (NRPS) gene clusters that are involved in the generation of ferrichrome and ferrichrome A, while the terpenes gene cluster is composed of three unknown function genes and seven biosynthesis related genes.

**Conclusions:**

As a destructive pathogen to sugar industry, the *S. scitamineum* genome will facilitate future research on the genomic basis and the pathogenic mechanisms of sugarcane smut.

**Electronic supplementary material:**

The online version of this article (doi:10.1186/1471-2164-15-996) contains supplementary material, which is available to authorized users.

## Background

Sugarcane belongs to the *Saccharum* genus of the Poaceae family (also called Gramineae or true grasses), an economically important seed plant family that includes maize, wheat, rice, sorghum, and many other forage crops. Sugarcane is the world’s largest crop. In 2012, the Food and Agriculture Organization estimates that it was cultivated in 101 countries, on approximately 26.1 million hectares, and with a worldwide harvest of 1.83 billion tons. The 10-year trend for sugarcane production has been upward. Sugarcane is not only a cash crop, but it can also be used as livestock fodder. The main product of sugarcane is sucrose, which accounts for 80% of all sugar produced in the world and 92% of that in China. It is also used as raw material in human food industries (molasses, rum, bagasse etc.) and is fermented to produce sugarcane ethanol. Sugarcane ethanol represents 40% of the world’s total ethanol fuel.

The increasing world demand for sugar and sugarcane ethanol keeps driving the development of sugarcane agriculture. The average yield of cane stalk is 60-70 tons per hectare per year. However, this figure can vary between 30 and 180 tons per hectare depending on knowledge and crop management approaches. During the production period, a sugarcane crop is sensitive to several biotic and abiotic factors, including ‘sugarcane smut’ which is a disease caused by the fungus *Sporisorium scitamineum*. Sugarcane smut has been an increasing large problem in almost all countries where sugarcane is grown. It was first reported in 1877 in the Natal region of South Africa [1]. Sugarcane smut can cause substantial losses in cane yield and sugar content in susceptible varieties. Losses can range from 30% to total crop failure, and the disease even leads to variety elimination due to susceptibility to this fungus [2].

The smuts are multicellular fungi-in spite of the unicellular yeast phase, which are characterized by their large numbers of dark, thick-walled and dust-like teliospores. *Ustilago*, *Sporisorium* and *Macalpinomyces* are the three genera of smut fungi (subphylum Ustilaginomycotina). These three genera are comprised of approximately 530 described species that all infect grasses [3]. Compared to the ambiguous position of *Macalpinomyces*, previous studies have demonstrated that *Ustilago* and *Sporisorium* together form a monophyletic group with the Ustilaginomycotina [4, 5]. The close relationship between *Ustilago* and *Sporisorium* can also be evidenced by the misclassification of *S. scitamineum* as *U. scitaminea*. Generally, *Ustilago* infects all aerial parts of the plant and rapidly forms galls or tumors filled with spores. In contrast, *Sporisorium* infects young seedlings, remains asymptomatic, and grows systemically until the emergence of mass of sooty spores.

**Figure 1:**
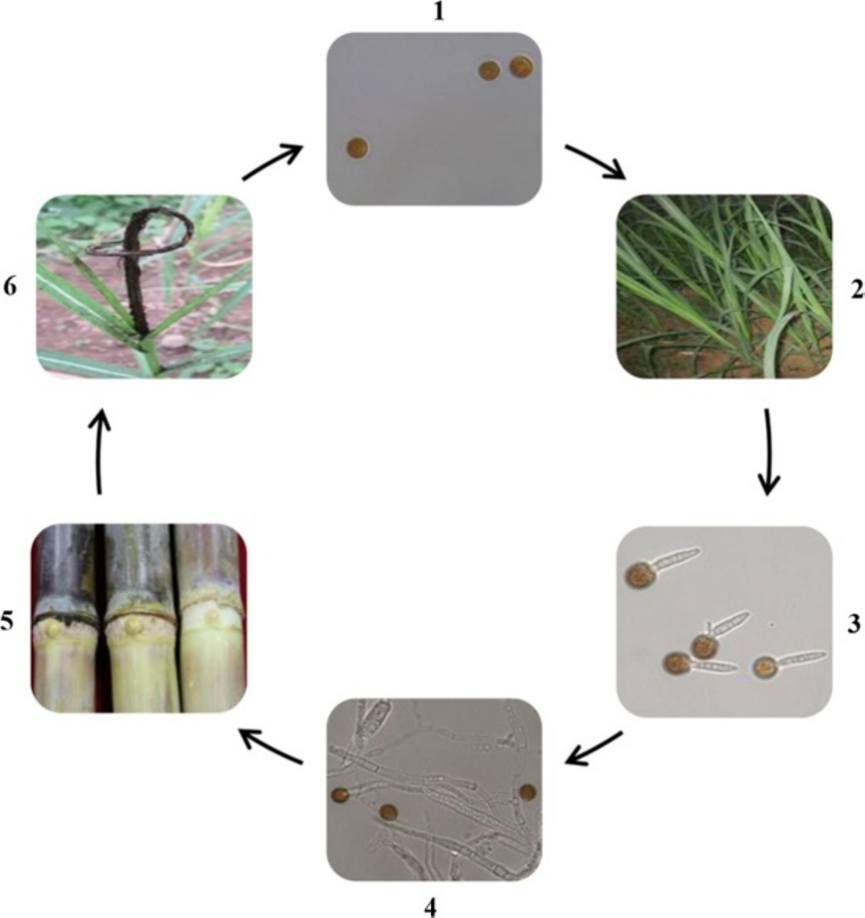
**Infection process of** ***Sporisorium scitamineum*** **on sugarcane.** 1, Teliospore; 2, Soil or sugarcane plants contaminated with *S. scitamineum*; 3, Teliospore germination; 4, The formation of infection hyphae; 5, Hypha infection in sugarcane meristem and growth point; 6, The formation of disease panicle.

*S. scitamineum,* previously known as *U. scitaminea,* is a basidiomycete and the causal agent of the sugarcane smut disease. It is among the most important phytopathogenic fungi in sugarcane and has significant impact on sugarcane production. This pathogen results in thin stalks, stunted plant growth, and an outgrowth of fungus of the stalk on the cane known as culmicolous [2] (Figure 1). The infected plants have increased tillering that results in more slender and much weaker leaves. The most recognizable characteristic of this disease is a black or gray outgrowth of a whip-like structure that is referred to as a “smut whip” [1] (Figure 1). These structures emerge from the terminal bud or from the lateral shoots of infected stalks and are composed of both plant and fungal tissues (Figure 1). Within these whip-like structures are millions of teliospores that are responsible for the quick dissemination of the disease. Teliospores germinate and undergo meiosis to form four haploid basidiospores. However, haploid cells are not infective and only the dikaryotic hyphae formed by fusion of compatible sporidia can infect the host. It is assumed that developing varieties of sugarcane resistant to sugarcane smut is the only economical method for control of this disease and the best course of action for management. During the selection process of sugarcane breeding, breeders have to abandon the development of a cross combination once smut disease appears in any of the progeny.

Despite the high worldwide losses in cane yield and sucrose caused by *S. scitamineum*, a limited amount of information is available about its genome structure and pathogenic mechanisms. A better understanding of the mechanisms of smut fungus-sugarcane interactions is necessary for the development and deployment of more effective and durable resistant cultivars. However, sugarcane smut fungi are biotrophic pathogens that cannot be cultured on artificial media. Most of their life stages, including urediniospores that are commonly used for aetiological and evolutionary biology studies, are dikaryotic. Therefore, it is extremely difficult to conduct molecular studies and functional characterizations of genes in sugarcane smut fungus. Besides its biological features, the genome is especially interesting because this is the first fungus important to the sugarcane industry to be completely sequenced. The insights from the three sequenced genomes of the biotrophic fungal plant pathogens, *Ustilago maydis* [6] and *Sporisorium reilianum* [7] in maize and *Ustilago hordei* [8] in barley, have highlighted the power of comparative genomics of closely related species for identification of virulence determinants and will no doubt facilitate the elucidation of the pathogenic mechanisms of sugarcane smut.

In the present study, we investigate the pathogenic mechanisms of sugarcane smut by sequencing, assembling, and annotating the genome of the smut fungus *S. scitamineum*. A comparative genomics study with the three related fungi *U. maydis*, *S. reilianum* and *U. hordei* was also conducted. This is the first high-quality genome sequence of *S. scitamineum* and is also the first reported genome of sugarcane fungi. This study may serve as a model for studying the pathogenic mechanisms in sugarcane, and this study provides knowledge for improving the sugar yield in sugarcane agriculture.

## Results and discussion

### *De novo*genome sequencing and assembly

The genome of *S. scitamineum* was sequenced using a whole-genome shotgun approach. A total of 6.32 Gb of raw sequences were generated from the Illumina Hiseq2000 platform (Additional file 1: Table S1) at BGI-ShenZhen. The total assembly size of the genome of *S. scitamineum* is 19.8 Mb which was assembled into 321 contigs and 58 scaffolds (Table 1). According to the k-mer analysis (Additional file 2: Figure S1a), the evaluated genome size of *S. scitamineum* is 25.9 Mb, larger than the assembled one, on account of repeat sequences in the genome. In Additional file 2: Figure S1b, the GC content and depth is normal which illustrates that the genome of *S. scitamineum* has less heterozygosity. The genome properties of *S. scitamineum*, including genome size, and GC content, is similar to the species closely related to smut fungi, especially *U. maydis*.

**Table 1:** ***Sporisorium scitamineum*** **genome statistics compared to that of other sequenced smut fungi**

Genome specifics	***Ss***	***Uh***	***Um***	***Sr***
Assembly statistics				
Total contig length (Mb)	19.8	20.7	19.7	18.2
Total number of contigs	321	2,953	275	915
Total scaffold length (Mb)	19.9	21.2	19.7	18.5
Total number of scaffolds	58	713	275	45
Average base coverage (X)	213	20	10	20
N_50_ contig (kb)	41	15.8	127	36
N_50_ scaffold (kb)	884.0	307.7	127.0	772.3
GC content (%)	54.85	52.00	54.00	59.70
Coding sequence				
Percentage coding (%)	64.4	59.9	64.1	66.9
Average gene size (bp)	1,936	1,782	1,935	1,852
Average gene density (kb/gene)	2.98	2.91	3.01	2.73
Protein-coding genes	6,636	7,110	6,548	6,673
Exons	11,521	10,995	11,458	9,751
Average exon size (bp)	1,044	1,102	1,049	1,225
Exons/gene	1.74	1.55	1.75	1.46
Noncoding sequence				
Introns	4,885	3,161	4,920	3,078
Introns/gene	0.74	0.44	0.75	0.46
Average intron length (bp)	167	141	128	135
NCBI accession	JFOL01000000	CAGI01000001.1	NW_101241.1	FQ311472.1

N_50_ contig, Size in kilobases of ≥50% of the assembled contigs.

*Ss*, *Sporisorium scitamineum*; *Uh*, *Ustilago hordei*; *Um*, *Ustilago maydis*; *Sr, Sporisorium reilianum.*

### Genome annotation

We predicted 6,636 protein-coding genes with an average transcript size of 1,936 bp via *ab initio* and homology-based analyses (Additional file 1: Table S2). The gene density was 2.98 kb per gene, which is lower than that in *S. reilianum* (2.73 kb per gene) and similar to the other two smut pathogens *U. maydis* (3.01 kb per gene) and *U. hordei* (2.91 kb per gene). The absolute number of exons was largest in *S. scitamineum*, however, the average exon size was the shortest among these smut fungi. Interestingly, the whole genome character of *S. scitamineum* is most similar to *U. maydis* which belongs to a separate genus. Only 4,585 (69.09%), 2,922 (44.03%), 3,196 (48.16%), 2,985 (44.98%), and 6490 (97.80%) of the predicted genes had homologies with known functions in the SwissProt, Gene ontology (GO), Clusters of Orthologous Groups (COG), Kyoto Encyclopaedia of Genes and Genomes (KEGG), and non-redundant proteins (NR) databases, respectively. There were 1,353 genes common to all these classical protein databases (Additional file 1: Table S3).

With regard to non-coding genes, we identified, 150 tRNAs, 5 snRNAs, and 35 rRNA fragments from the assembly (Additional file 1: Table S4). Transposable elements play an important role in fungal pathogens [7]. The *S. scitamineum* genome is comprised of 1.58% repetitive elements and DNA transposons are dominant (Additional file 1: Table S5).

### Comparative genome analyses

**Figure 2:**
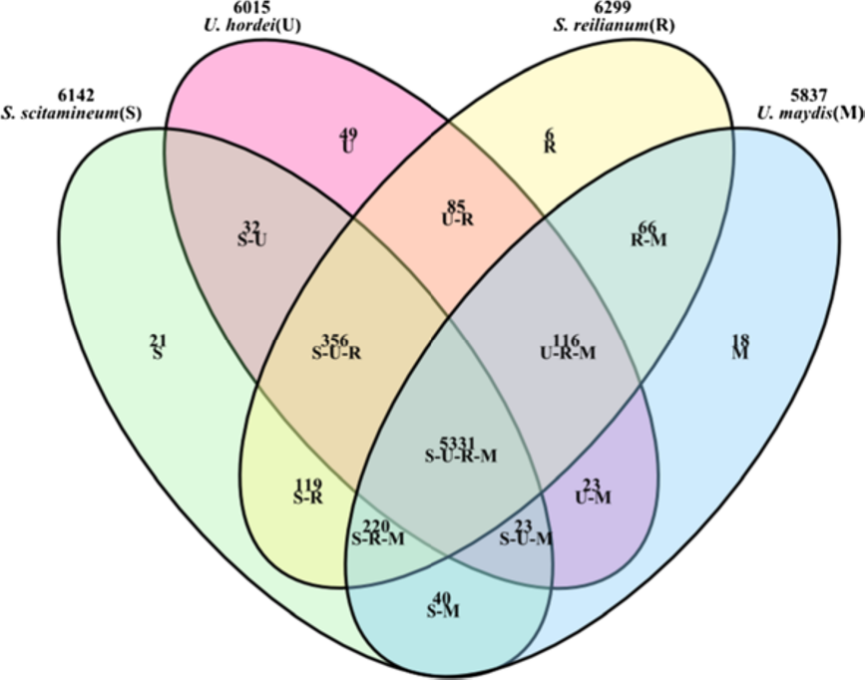
**Overlap among gene families for four smut fungi species.** This is the venn diagram used to compare the gene families of four smut fungi, four colors (green, red, yellow and blue) mean four gene family sets, and the overlap parts have the overlapping color. Numbers indicate the gene families in each comparison. S means the gene families specific to *S. scitamineum*, S-U means common gene families of *S. scitamineum* and *U. hordei*, S-U-R means common gene families of *S. scitamineum*, *U. hordei* and *S. reilianum*, S-U-R-M means common gene families of four smut fungi.

The homology between *S. scitamineum* and three other smut fungi was examined via gene families. There were 5,331 gene families shared among all four smut fungi, and there were 48 predicted genes in 21 gene families that appear to be unique to *S. scitamineum* (Figure 2). Among these unique genes, most have unknown functions. Twenty-seven genes did not receive hits in protein databases, and 11 genes annotated to NR even belong to hypothetical or uncharacterized protein encoding genes. These unique genes could act as a potential mine to explore. Main gene families, which have large gene members, included ATP-binding cassette (ABC) transporter, oligopeptide transporter, P-type ATPase (transport), helicase and integrase (DNA binding), and heat shock protein 70 family.

**Figure 3:**
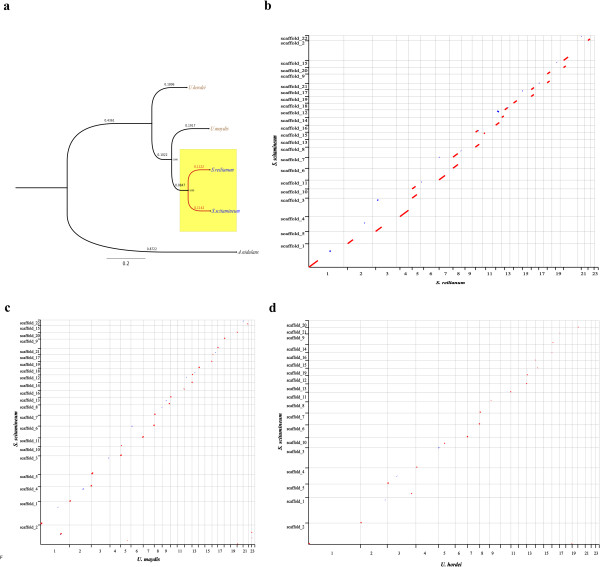
**Taxonomic placement of** ***Sporisorium scitamineum*** **and genome synteny with** ***S. reilianum*** **,** ***Ustilago maydis,*** **and** ***U. hordei*** **. (a)** Evolutionary placing of *S. scitamineum* in relation to other smut fungi and *A. nidulans.* Numbers on the branch indicate branch length. Numbers for the internal node indicate bootstrap value; **(b)** Dot plot of the synteny occurring between the assembled chromosomes of *S. reilianum* and the assembled scaffolds of *S. scitamineum*; **(c)** Dot plot of the synteny occurring between the assembled chromosomes of *U. maydis* and the assembled scaffolds of *S. scitamineum*; **(d)** Dot plot of the synteny occurring between the assembled chromosomes of *U. hordei* and the assembled scaffolds of *S. scitamineum*.

A phylogenetic analysis revealed the evolutionary relationship among smut fungi and positioning of *S. scitamineum*. It showed that *S. scitamineum* and *S. reilianum* are sister taxa and that *U. maydis* branches earlier in the evolutionary history (Figure 3a). The genomes of *U. maydis* [6], *S. reilianum* [7], and *U. hordei* [8] are all organized in 23 chromosomes. They could be used as references to map the genome of *S. scitamineum*. Our results exhibited a remarkably higher degree of synteny between *S. scitamineum* and *S. reilianum* than that between *S. scitamineum* and *U. maydis* or *U. hordei* (Figure 3b,c,d). Meanwhile, the fact that *S. scitamineum* and *S. reilianum* share most common gene families could support the relationship of these two species. We conclude that the chromosome number of *S. scitamineum* is possibly twenty-three based on the high synteny between *S. scitamineum* and *S. reilianum* and the chromosome numbers of the three smut fungi.

### Characterization of mating type loci in *S. scitamineum*

For mating to occur, two haploid cells of different mating-type need to recognize each other and fuse to form the infectious dikaryon. Mating is regulated by two loci, *a* and *b*, which harbor conserved genes. At the *a* locus, these genes encode pheromones and pheromone receptors while at the *b* locus two subunits of a heterodimeric transcription factor are encoded [9].

**Figure 4:**
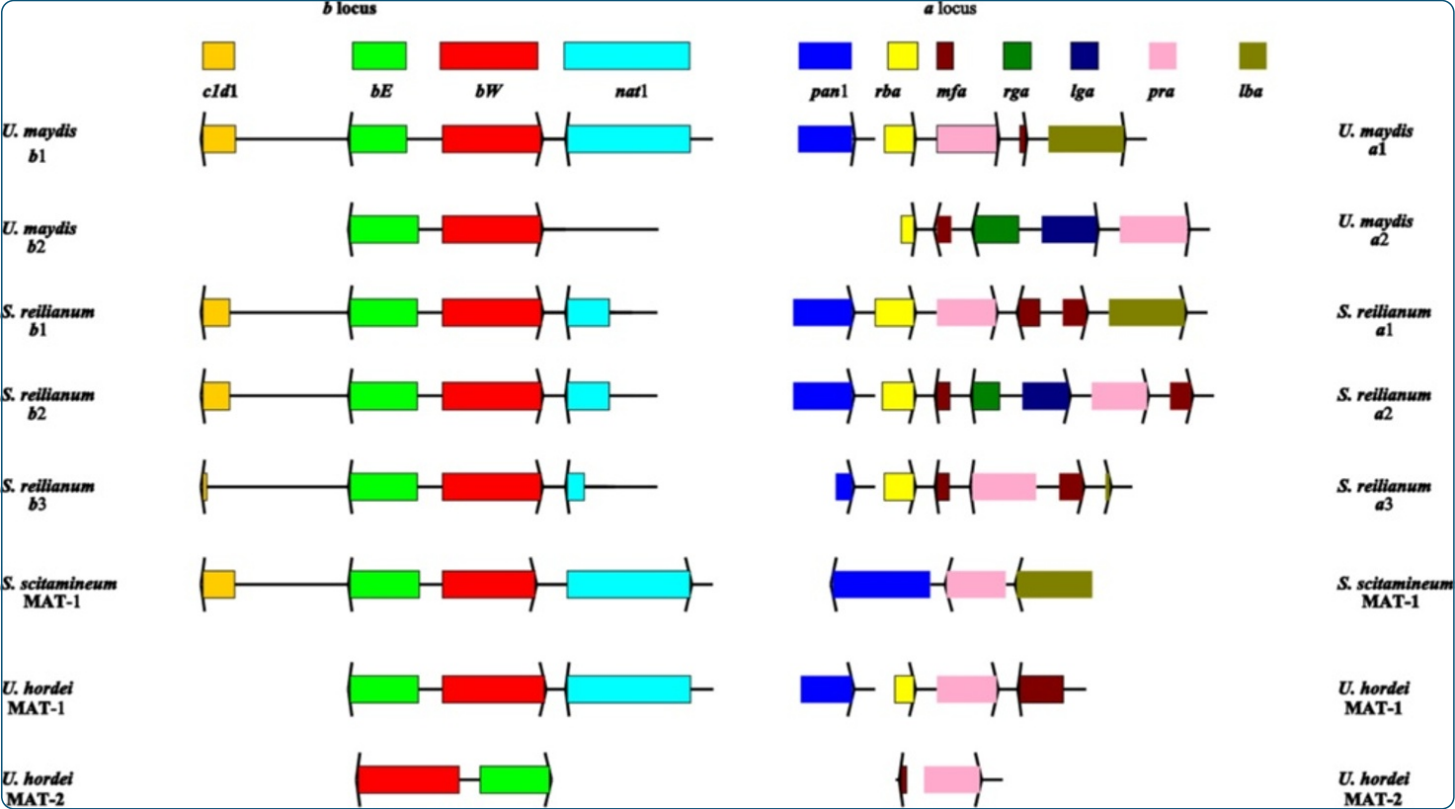
**Genetic organization of the mating-type loci of smut fungi.** Genes are indicated by arrows with the arrow denoting the direction of transcription. Related genes are denoted by the same color and respective gene functions are explained in the lower part of the Figure. *indicates that the relative order and orientation of these genes have not been determined. In the tetrapolar species, *U. maydis* and *S. reilianum*, the *a* and *b* specific sequences reside on different chromosomes, while they are linked by spacer regions (which are not drawn to scale and whose length is indicated) in the bipolar species *U. hordei* and *S. scitamineum*. The black bars on top of the figure indicate the regions of the *b* locus, which covers the two homeodomain protein genes *bE* and *bW*, and the *a* locus (that expands to different length in the different loci, indicated by a broken line) from the *lba* gene to the *rba* gene. Sequence information was obtained from the following Accession Numbers: AF043940, AM118080, AF184070, AF184069, Z18531, AJ884588, AJ884583, AJ884590, AJ884585, AJ884589, AJ884584, U37796, M84182, AACP01000083 and AACP01000013.

The bipolar species *S. scitamineum* and *U. hordei* as well as the tetrapolar species *U. maydis* and *S. reilianum* possess one divergently transcribed gene pair that encode the homeodomain proteins bE (HD1) and bW (HD2). The MAT-1 locus, gene order, orientation, as well as the genomic context are conserved in the *b* mating-type genes except for the *U. hordei* MAT-2 locus (Figure 4). Interestingly, both *bE* and *bW* mating-type genes are present in the genomes of Ustilaginaceae including the two genera of *Ustilago* and *Sporisorium*.

In addition to the *b* mating-type complexes, smut fungi contain genes necessary for cell–cell recognition which are located in the *a* mating type loci. The detailed structure of these loci has been determined for both the MAT-1 alleles of *S. scitamineum* and *U. hordei*, an allele of *U. maydis,* and for all three alleles of *S. reilianum* (Figure 4). Both *U. maydis* and *U. hordei* have two alleles of an *a* mating system with one pheromone receptor (*pra*) and one functional pheromone gene (*mfa*) per locus.

In *S. scitamineum* and *U. hordei* which have a bipolar mating system, the *a* an*d b* loci are linked and the mating-type locus (MAT) segregates as one locus. However, in tetrapolar species such as *S. reilianum* and *U. maydis*, these genetic loci segregate independently [7]. In *S. scitamineum*, the *a* locus encodes a lipopeptide with pheromone and pheromone membrane receptor functions responsible for cell recognition and compatible hyphal fusion, whereas the *b* locus encodes transcription factors that control the expression of genes responsible for the maintenance of the dikaryotic hyphal growth in plants (Figure 4). During their life cycle, *S. scitamineum* presents two distinct monokaryotic and dikaryotic stages. The monokaryotic stage is marked by haploid cells that grow saprophytically and are not able to cause disease, while in the second phase, dikaryotic hyphae are formed by mating (sexual crossing) and are able to infect the host. The induction of the pathogenic program in *S. scitamineum* implies not only strong morphological changes (from yeast-like to hyphal) but also genetic changes (haploid to dikaryotic transition).

Evolution of bipolar mating in *S. scitamineum* may have been beneficial for the fungus because it promoted inbreeding and stabilization of the genome. The same process has been proved to be beneficial for the transposon elements (TEs). A study concluded that inbreeding helped fix TEs within a population in *U. hordei* [10]. In tetrapolar species, such as *U. maydis* and *S. reilianum*, outcrossing increases heterozygosity [7].

Overall, sequence analysis and comparison of the mating-type regions of tetrapolar and bipolar smut fungi revealed that they are not fundamentally different. Bipolar and tetrapolar smuts as well as related species contain the genes for these *a* and *b* mating-type complexes. In the bipolar species *S. scitamineum* and *U. hordei*, these mating-type complexes are encoded on the same chromosome and in a recombination-suppressed region ensuring genetic linkage.

### G-protein–coupled receptors

To successfully infect a host plant, plant pathogenic fungi and oomycetes must make appropriate responses to a variety of environmental cues, including the chemical and physical characteristics of the host plant surface. This is achieved via cell surface receptors that respond to external stimuli and relay that information into the cell. G protein-coupled receptors (GPCRs), which are characterized by having seven transmembrane helices, represent the largest family of cell surface receptors and are responsible for transducing extracellular signals into intracellular responses that involve complex intracellular-signaling networks [11–14]. The genomes of animals contain large numbers of GPCRs, while the genomes of the model ascomycete yeast species *Saccharomyces cerevisiae* and *Schizosaccharomyces pombe* contain only three and four GPCRs [11], respectively. However, a significant number of putative GPCRs have been demonstrated in the analysis of the genomes of filamentous phytopathogenic ascomycete fungi, as many as 61 have been reported in the rice blast fungus *Magnaporthe grisea* and 84 have been demonstrated in the head blight pathogen *Fusarium graminearum* [12, 13]. Previous researches also revealed that GPCRs are extremely diverse in sequence and function. Constraints in structure prediction impaired research on these proteins for a long time [11–16], highlighting the necessity of identifying and characterizing far more GPCRs.

Here we present an exploration of the genomes of 12 fungi, the *S. scitamineum* plus other 11 fungi, and identify members of the G protein-coupled receptor family from the entire deduced proteomes. It demonstrated that *S. scitamineum* possesses only 6 GPCRs, which are grouped into 5 classes (Table 2). This total set of analyses also resulted in the identification of 7 and 5 putative GPCRs in *S. reilianum* and *U. maydis* respectively, compared to the 10 predicted GPCRs in the *U. hordei* (Table 2)*.* This suggests that the number of GPCRs in *S. scitamineum* is closer to those of *S. reilianum* and *U. maydis* than that of *U. hordei*. This is in accordance to the phylogeny of these fungi based on comparative genome analyses showing in Figure 3. PTH11, a cell-surface integral membrane protein required for pathogenicity [14], has seven transmembrane regions and an amino-terminal extracellular cysteine-rich EGF-like domain (CFEM domain). The PTH11-like GPCR is a PHI protein shown to regulate *M. grisea* appressorium differentiation in response to the plant surface [14]. We demonstrated that no PTH11-like GPCRs were found in the genome of all the four sequenced smut fungi, including *S. scitamineum, S. reilianum*, *U. maydis* and *U. hordei* (Table 2).

**Table 2:** **Classification of putative GPCRs identified among various plant pathogen fungi**

GPCR class	Biotroph				Obligate_biotroph		Hemibiotroph		Necrotroph				Characteristics/domains
	***Ss***	***Uh***	***Um***	***Sr***	***Mlp***	***Pgt***	***Mo***	***Fg***	***Vd***	***Fo***	***Fv***	***Nh***	
I (pheromone receptors)	0	0	0	0	0	0	1	1	1	1	1	1	STE2-type
II (pheromone receptors)	0	1	1	1	4	3	1	1	0	1	1	1	STE3-type
III (related to *A. nidulan* GprC, GprD, and GprE)	1	1	0	1	3	3	5	2	3	5	4	3	Git3 (G protein-coupled glucose receptor) domain
IV (nitrogen sensors)	1	3	1	1	0	2	2	2	2	3	2	2	PQ-loops
V (cAMP receptor-like)	0	0	0	0	0	0	2	4	2	8	5	4	Secretin-family/Dicty_CAR domain
VI (GPCRs containing RGS domain)	0	0	0	0	0	0	2	0	1	2	2	3	RGS-domain
VII (related to rat growth hormone releasing factor)	0	0	0	0	0	0	1	0	1	3	2	1	Secretin-like
VIII (related to human steroid receptor mPR)	0	0	0	0	2	2	3	1	2	2	3	2	HlyIII-superfamily
IX (microbial opsins)	2	2	2	2	2	1	1	3	2	6	6	5	Bac_rhodopsin
X (similar to PTM1)	1	1	1	1	1	1	1	1	1	1	1	1	Lung_7TM superfamily
XI (similar to GPCR89)	0	1	0	0	0	0	0	0	1	0	0	0	ABA_GPCR domain
XII (family C-like GPCRs)	0	0	0	0	0	0	2	1	1	3	1	1	
XIII (related to GPR11 of *P. sojae*)	1	1	0	1	1	5	2	1	0	3	1	1	DUF300 superfamily
PTH11-like	0	0	0	0	0	0	9	2	3	10	5	6	Related to *M. grisea* PTH11 receptor

*Ss*, *Sporisorium scitamineum*; *Uh*, *Ustilago hordei*; *Um*, *Ustilago maydis*; *Sr*, *Sporisorium reilianum*; *Mlp*, *Melampsora laricis-populina*; *Pgt*, *Puccinia graminis* f. sp. *tritici*; *Mo*, *Magnaporthe oryzae*; *Fg*, *Fusarium graminearum*; *Vd*, *Verticillium dahliae*; *Fo*, *Fusarium oxysporum*; *Fv*, *Fusarium verticillioides*; *Nh*, *Nectria haematococca*.

*S. scitamineum* may acquire the ability to respond to a variety of environmental cues using different GPCRs that activate conserved intracellular signaling pathways and give new triggers to response systems. The identification and characterization of GPCRs will provide insights into how *S. scitamineum* communicates with its environment and senses the presence of intracellular signaling.

### Carbohydrate degrading enzymes

Phytopathogenic fungi secrete a cocktail of hydrolytic enzymes (including carbohydrateactive enzymes, CAZymes) for degrading the plant cell wall and penetrating into the host tissue. The *S. scitamineum* genome encoded 867 putative CAZymes including 337 glycoside hydrolases (GH), 253 glycosyltransferases (GT), 92 carbohydrate esterases (CE), 181 carbohydrate binding modules (CBM), and 4 polysaccharide lyases (PL) comprising more than 130 distinct families (Table 3). There were 103 genes that could be annotated by the five databases utilized (SwissProt, GO, KEGG, COG and NR), among which 18 genes are putative plant cell wall hydrolytic enzymes (GH43, GH51, GH78 and PL4 families) [17]. Most of these enzymes belong to GH78 family (13 genes) and are involved in pectin degradation. Interestingly, pectin is not the main part of the sugarcane cell wall [18]. In terms of carbohydrates, the larger amount of hemicelluloses that comprise up to 50% of sugarcane cell wall [18] could be broken down by the GH43 family (SmutADNA4_GLEAN_10001516, SmutADNA4_GLEAN_10004227) coding xyloglucan:xyloglucosyltransferase and beta;-xylosidase as well as the GH51 family (SmutADNA4_GLEAN_10004371, SmutADNA4_GLEAN_10005166) coding alpha;-L-arabinofuranosidase and alpha-L-arabinofuranosidase. The minimal set of hemicellulases found in *S. scitamineum* seems perfectly in line with its biotrophic lifestyle, in which damage to the host is minimized and the release of cell wall fragments, which often trigger plant defense responses, is avoided [6].

**Table 3:** **Comparative analysis of CAZyme family among various plant pathogen fungi**

		CBM	CE	GH	GT	PL	Total	PCW
**Biotroph**	*Ss*	181	92	337	253	4	867	68
	*Uh*	178	83	451	276	5	993	122
	*Um*	181	103	346	262	3	895	53
	*Sr*	183	98	339	248	4	872	54
**Obligate_biotroph**	*Mlp*	287	141	448	292	7	1,175	124
	*Pgt*	260	93	415	309	6	1,083	85
**Hemibiotroph**	*Mo*	411	177	701	399	9	1,697	125
	*Fg*	373	224	821	439	28	1,885	158
**Necrotroph**	*Vd*	351	182	757	361	45	1,696	202
	*Fo*	669	421	1,587	881	37	3,595	351
	*Fv*	595	331	1,294	668	35	2,923	238
	*Nh*	452	334	1,230	527	46	2,589	254

*Ss*, *Sporisorium scitamineum*; *Uh*, *Ustilago hordei*; *Um*, *Ustilago maydis*; *Sr*, *Sporisorium reilianum*; *Mlp*, *Melampsora laricis-populina*; *Pgt*, *Puccinia graminis* f. sp. *tritici*; *Mo*, *Magnaporthe oryzae*; *Fg*, *Fusarium graminearum*; *Vd*, *Verticillium dahliae*; *Fo*, *Fusarium oxysporum*; *Fv*, *Fusarium verticillioides*; *Nh*, *Nectria haematococca*. CBM, carbohydrate binding module; CE, carbohydrate esterase; GH, glycoside hydrolases; GT, glycosyltransferase; PL, polysaccharide lyase; PCW, plant cell wall degradation related enzyme.

**Figure 5:**
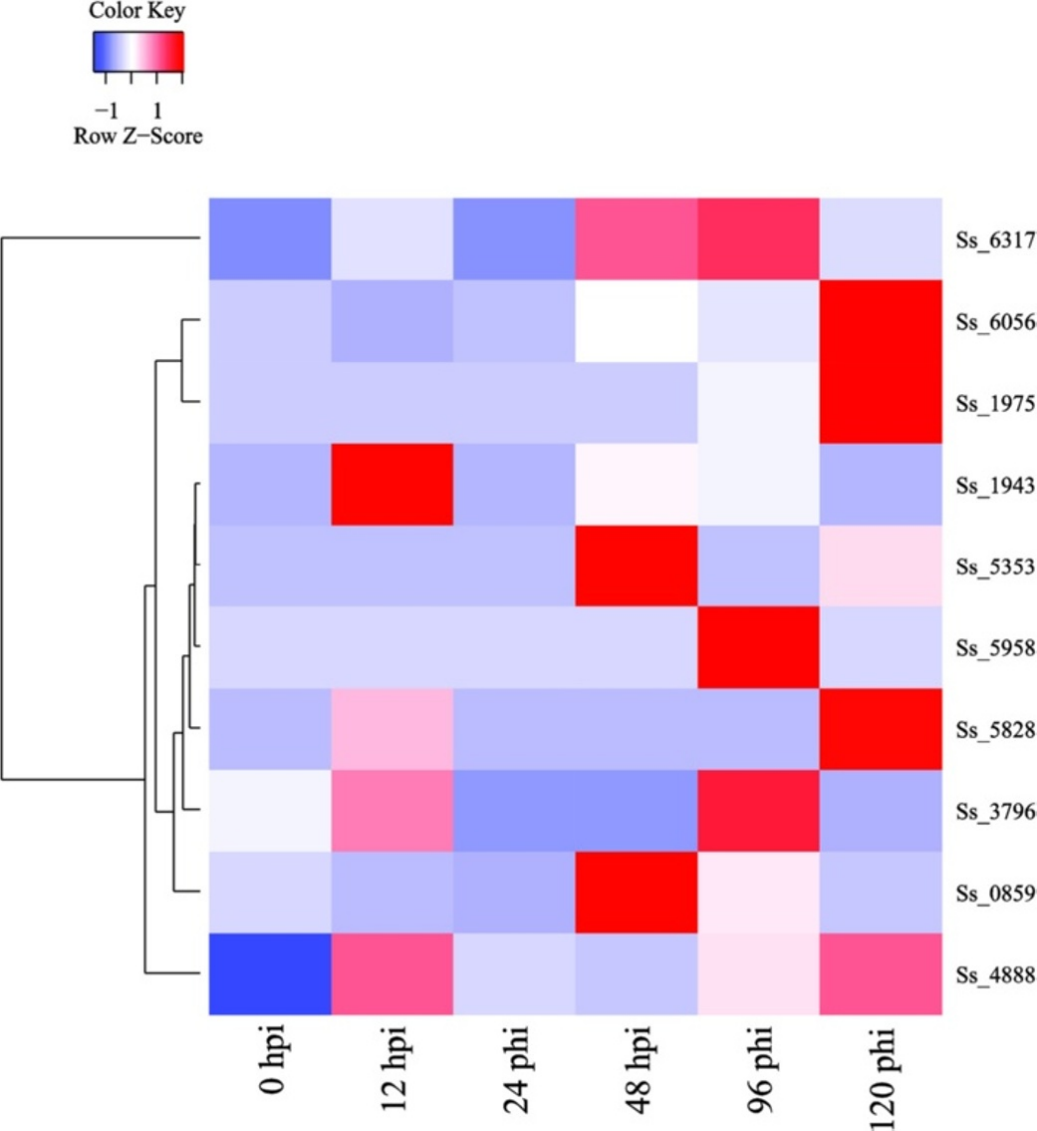
**The expression pattern of candidate genes for encoding pant cell wall degradation enzymes of** ***Sporisorium scitamineum*** **at six stages in the infection process.** These infection stages includes six time points during the course of infection (the 0-, 12-, 24-, 48-, 96-, and 120-hpi). The number on the left side, such as Ss 6317, means gene ID. The color scale indicates relative RT-qPCR expression.

Real-time quantitative PCR (RT-qPCR) analysis was performed to examine the expression of these 18 genes during the course of infection (0, 12, 24, 48, 96 and 120 h post-inoculation (hpi)). The result suggests that there are only ten genes of the GH78 family were expressed and half of them expressed in the initial stage of infection (Figure 5 and Additional file 1: Table S6). One gene (SmutADNA4_GLEAN_10004888) encoding alpha;-L-rhamnosidase (EC 3.2.1.40), expressed in all stages and reached the highest expression at 12 hpi. It is quite a surprise that no expression was detected for the GH43, GH51 and PL4 families, while the gene expression profiles of the GH78 family are distinct at different infection time points. The regulated expression of ten members of the GH78 family suggests that these key enzymes have a higher activity and play an important role during host infection. However, the expression of a specific gene encoding degradation-associated enzymes should cause damage to the sugarcane plant during infection only at a certain point.

### Biotrophic properties and utilization of carbon and nitrogen substrates

In order to grow and propagate, phytopathogens need to take up nutrients, mainly ammonium and sulfate, from their parasitic host. Obligate biotrophic pathogens which include powdery mildews, rust fungi, downy mildews and *Albugo* spp., lack genes encoding for particular classes of plant cell wall hydrolases and for various metabolic processes of products, such as nitrates and proteins involved in sulfate assimilation [19]. These losses are interpreted as convergent adaptations.

**Figure 6:**
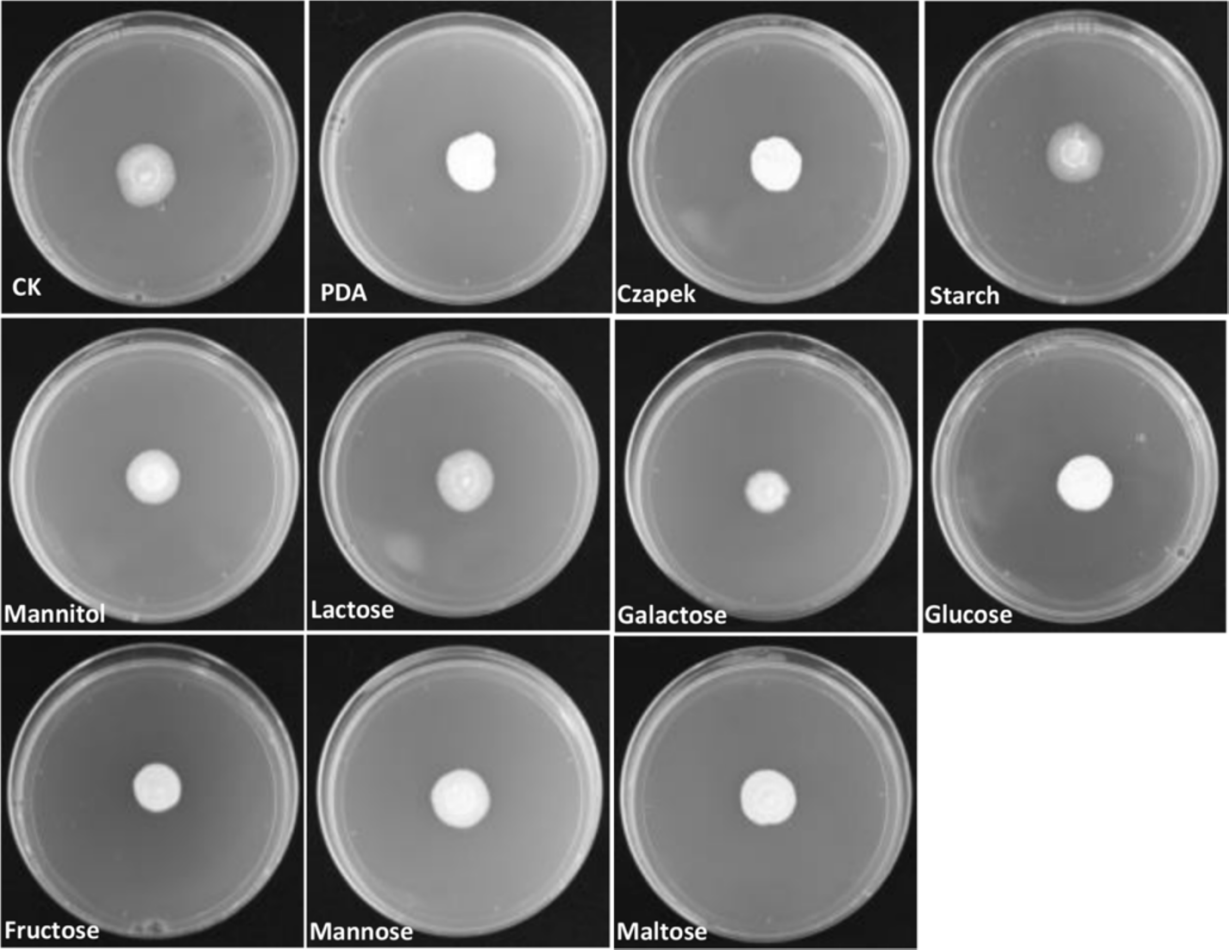
**Comparative growth profiling of** ***Sporisorium scitamineum*** **on various carbon substrates.** This figure is used to explain that *S. scitamineum* can utilize all the eight carbon substrates without significant differences.

**Figure 7:**
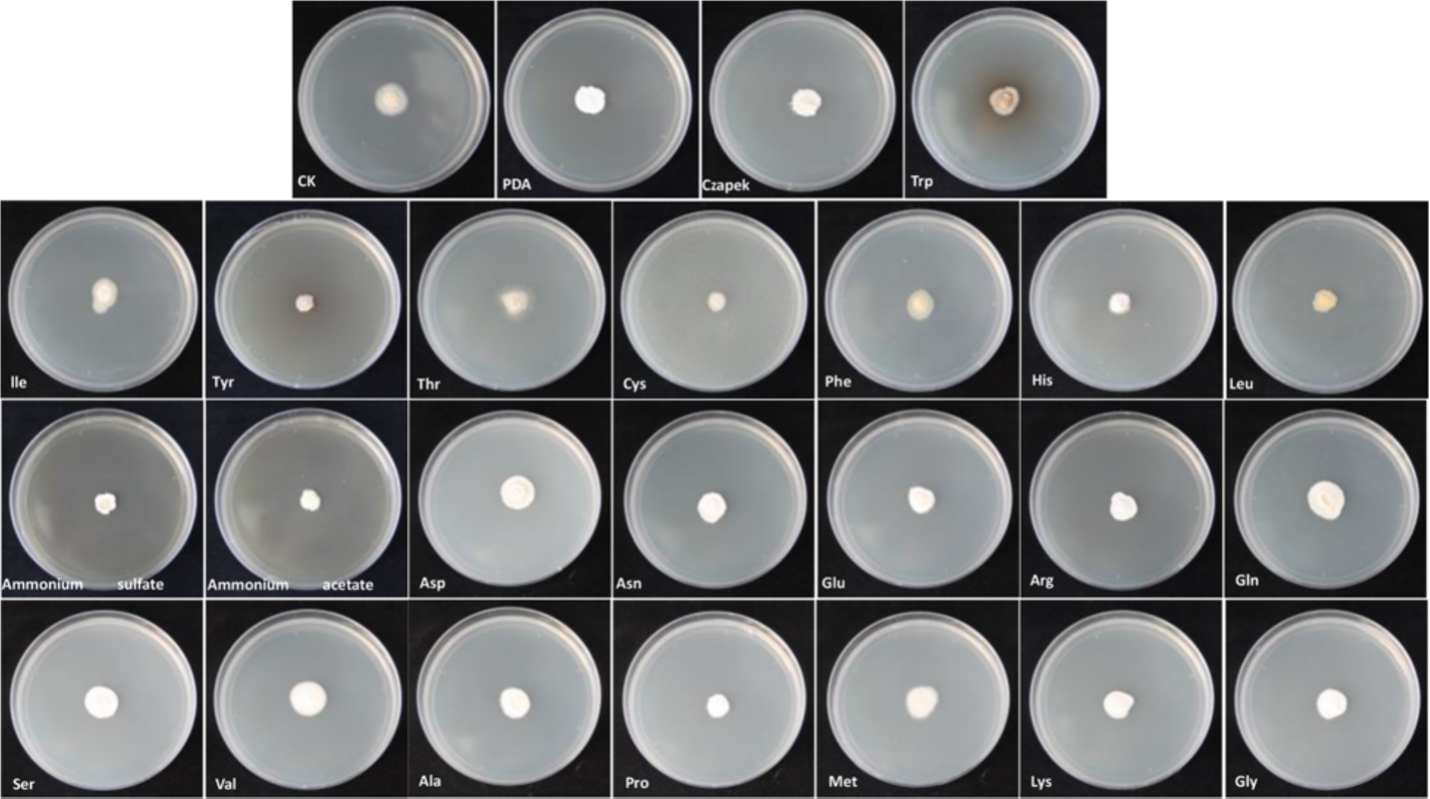
**Comparative growth profiling of** ***Sporisorium scitamineum*** **on various nitrogen substrates.** This figure is used to explain that *S. scitamineum* can uptake a broad range of nitrogen sources, including sodium nitrate, ammonium sulfate, ammonium acetate, and all the twenty kinds of amino acids, without significant differences.

We anticipated that growth profiles could illuminate correlations between a pathogen and its respective gene complements. Here, we present the comparison of growth on eight carbon and 23 nitrogen substrates. As shown in Figure 6*, S. scitamineum* can utilize all the eight carbon substrates, which is similar to the assimilation of *Cladosporium fulvum* and *Dothistroma septosporum* on carbon substrates [20]. In general, *S. scitamineum* grows more slowly on the media with carbon substrates of mannitol, lactose, and galactose than on the media with carbon substrates of glucose, fructose, mannose or maltose. The pattern was reversed when the minimal control medium Czapek was also present. The pronounced growth of *S. scitamineum* on the medium with sucrose is particularly striking and suggests that the fungus can utilize sucrose available in apoplastic fluid during its early biotrophic colonization phase. The result is in accordance with previous reports on *C. fulvum* [20]. The present study also showed that *S. scitamineum* can uptake a broad range of nitrogen sources, including sodium nitrate, ammonium sulfate, ammonium acetate, and all the twenty kinds of amino acids (Figure 7). The same phenomenon of extensive use of nitrogen sources has also been witnessed in *Aspergillus* [21] and this emphasizes the impact of nutritional flexibility on fungal pathogenicity. The above-mentioned carbohydrate and nitrogen metabolism pathways including glycolysis, fructose and mannose metabolism, sucrose metabolism, amino acid metabolism, as well as nitrate and sulphate assimilation, are present in the genome of *S. scitamineum*. However, due to the non-uniform growth habit of *S. scitamineum* on these media, further information is needed for conclusions about its ability to utilize carbon and nitrogen substrates. In addition, more experiments must be conducted to support the hypothesis that transporter genes are responsible for the uptake of carbon and nitrogen from plant cells and to confirm the assumption that the broad range of utilization in carbon and nitrogen substrates should result in the wide adaptability of *S. scitamineum* to various environmental conditions.

The smut fungus *U. maydis* has a reduced set of genes encoding plant cell wall hydrolytic enzymes, presumably as an adaptation to its biotrophic lifestyle [6]. A similar schematic representation of CAZyme is found in the *S. scitamineum* with the approximate types and sum (Table 3) as typical biotrophic fungi. Biotrophic fungi tend to have fewer plant cell wall degrading enzymes (PCW) than necrotrophic and hemibiotrophic fungi, and the necrotrophic fungi have the most abundant numbers of PCWs.

### Virulence associated genes

**Figure 8:**
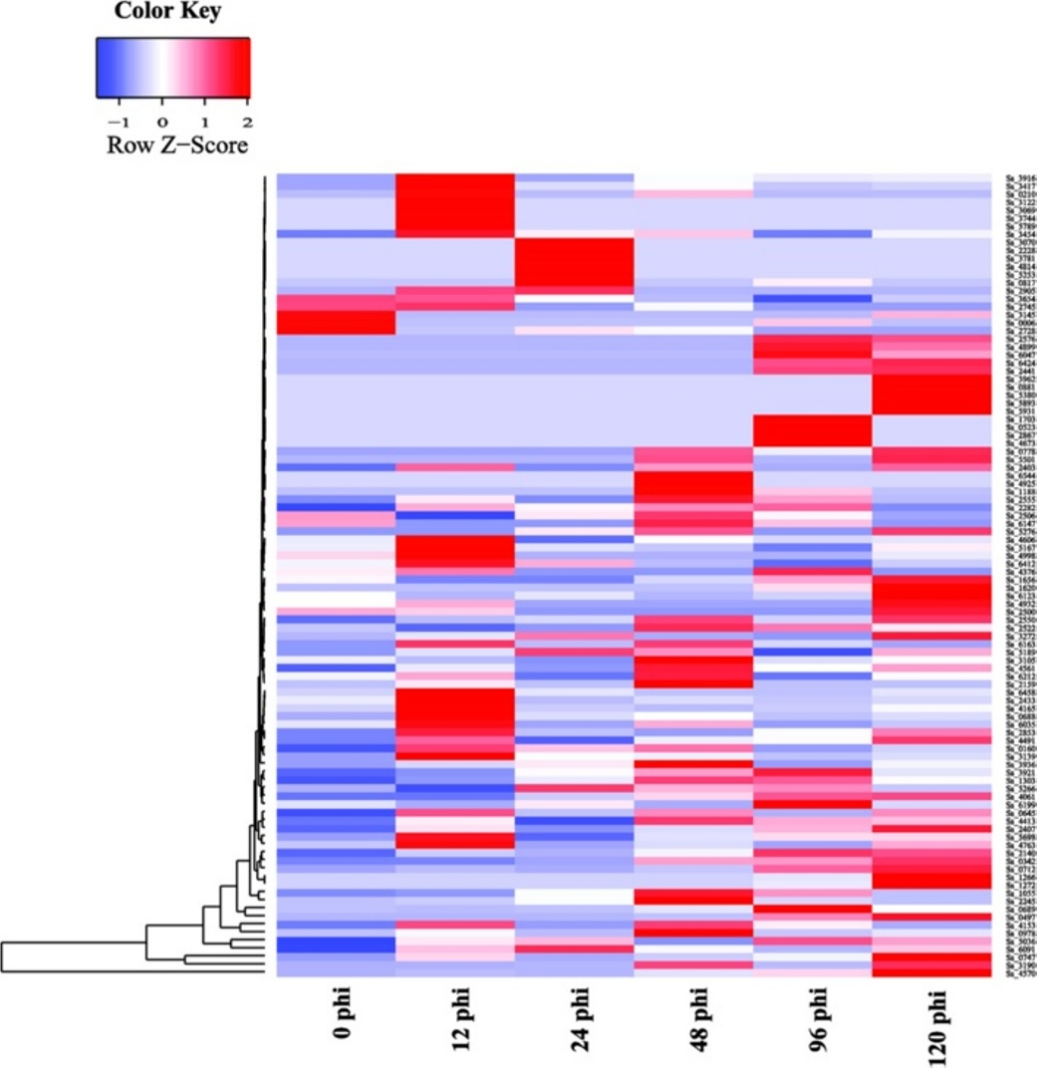
**The expression pattern of candidate virulence associated genes of** ***Sporisorium scitamineum*** **at six stages in the infection process.** These infection stages includes six time points during the course of infection (the 0-, 12-, 24-, 48-, 96-, and 120-hpi). The number on the left side, such as Ss 3916, means gene ID. The color scale indicates relative RT-qPCR expression.

As a biotrophic pathogenic fungus, *S. scitamineum* is expected to possess a number of pathogen-host interaction genes. The genome was searched using the pathogen-host interaction database (PHI-base) [22] and 1,091 putative PHI genes were identified. After filtering the genes from bacteria plant pathogens and animal or human pathogens, there were 192 genes left that could be commonly annotated by five databases (SwissProt, GO, KEGG, COG and NR) as the candidate virulent factor. These 192 genes play diverse roles in redox of short-chain compounds, cell wall breakdown energy metabolism and transport (Additional file 1: Table S7). RT-qPCR analysis was performed to examine the expression profiles of these 192 genes during the course of infection (0, 12, 24, 48, 96 and 120 hpi) and 52% of them expressed in the process of infection (Figure 8 and Additional file 1: Table S7). The result suggests that 31 genes were expressed in all the stages and mainly encoded energy metabolism and redox of short-chain compound related enzymes (Figure 8 and Additional file 1: Table S7). The gene expression of SmutADNA4_GLEAN_10004570 which encodes peptidyl-prolyl cis-trans isomerase B (cyclophilin B) continued to increase at all stages and reached its maximum at 120 hpi (a 388 fold change). Cyclophilin B possesses cis-trans peptidyl-prolyl isomerase activity and probably is an important determinant of the virulence of pathogenesis [23]. The function of these virulence associated genes could be elucidated by gene disruption or complementation.

### CSEPs and their expression

**Figure 9:**
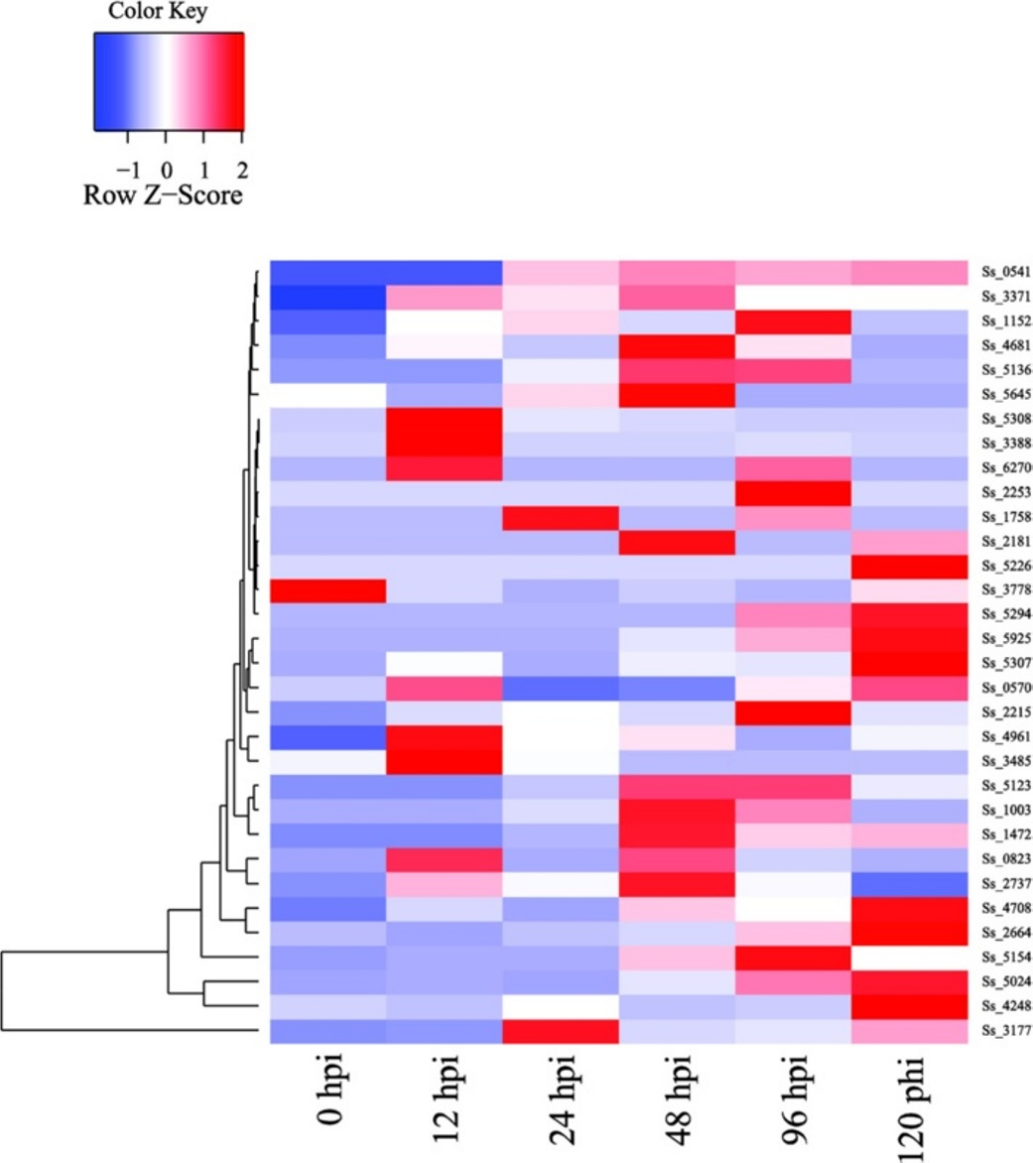
**The expression pattern of genes coding for secreted effector proteins of** ***Sporisorium scitamineum*** **at six stages in the infection process.** These infection stages includes six time points during the course of infection (the 0-, 12-, 24-, 48-, 96-, and 120-hpi). The number on the left side, such as Ss 0541, means gene ID. The color scale indicates relative RT-qPCR expression.

Recent researches have revealed the extensive repertoires of pathogen effectors and their various functions. The pathogenicity of *S. scitamineum* has been studied. However, no effector has been reported prior to our analysis. Effectors have been screened via *de novo* analysis from the gene set according to the description of Olof Emanuelsson *et al*. in the Nature PROTOCOL [24]. There are 68 proteins (Figure 9 and Additional file 1: Table S8) that comprise the Candidates for Secreted Effector Proteins (CSEPs) of *S. scitamineum* which are clustered into 10 families (21 CSEPs) in Table 4. Most of CSEPs in these 10 families have more than 2.0% cysteine content and longer amino acids. Interestingly, there were just two CSEPs un-annotated to the NR database, however, 43 of 66 annotated proteins are conserved hypothetical proteins or uncharacterized proteins. Many of the functions of CSEPs are unknown in biotrophic phytopathogenic fungi, such as *U. maydis* [6] and *Blumeria graminis* [25]. RT-qPCR analysis revealed that 47% of CSEPs expressed in the infection process. Among them, 7 expressed at all stages while 12, 9, 5, 2, 2, and 1 CSEPs started to express at 0, 12, 24, 48, 96, and 120 hpi, respectively (Figure 9 and Additional file 1: Table S8). Four of the above-mentioned seven CSEPs belong to conserved hypothetical proteins with unknown function and did not receive hits from any protein database. The remaining three CSEPs have a clan GH-D domain (IPR000111), a catalytic domain (IPR008258), and a Barwin-related endoglucanase domain (IPR009009) as well as sections encoding glycoside hydrolase and lytic transglycosylase-like protein. These CSEPs are mainly involved in carbohydrate degradation. It is unclear how these CSEPs facilitate infection and/or trigger defense responses, how they are secreted and whether there is specific invasion structure to help the secretion. These conundrums require additional endeavors and deeper research.

**Table 4:** **Summary of the 10 CSEP families in the** ***Sporisorium scitamineum*** **genome**

Family	Number of members	C-term cysteine	Average cysteine content (%)	Average peptide length (aa)	IPR annotation	Expression in the infection process
1	3	Yes	1.6	500	IPR000560:Histidine phosphatase superfamily, clade-2	Yes
2	2	No	2.42	392	IPR013781:Glycoside hydrolase, subgroup, catalytic domain	Yes
3	2	Yes	1.95	385	IPR001283:Allergen V5/Tpx-1-related	Yes
4	2	No	2.03	615	IPR000111:Glycoside hydrolase, clan GH-D	Yes
5	2	No	2.2	567	IPR001563:Peptidase S10, serine carboxypeptidase	Yes
6	2	Yes	2.61	230	N/A	No
7	2	No	2.05	415	N/A	Yes
8	2	No	2.12	260	IPR009009:Barwin-related endoglucanase	No
9	2	No	2.31	346	N/A	No
10	2	Yes	1.76	398	IPR002509:Polysaccharide deacetylase	Yes

Protein essential during penetration 1 (Pep1), representing a novel effector for establishing a biotrophic interaction, has been identified and characterized in *U. maydis* [26, 27]. Pep1 is also found to be highly conserved in related pathogens such as *S. reilianum* and *U. hordei* [8, 26, 27]. However, all the above 68 CSEPs of *S. scitamineum* have no ortholog to Pep1. Accordingly, we expanded the search to all proteins and found that one protein encoded by SmutADNA4_GLEAN_10005329, has 176 amino acids with 65% identity to the *U. maydis* Pep1. It was demonstrated that all Pep1 proteins from the 4 smut fungi have an N-terminal secretion signal and four cysteine residues whose spacing is conserved as well as glycine-rich C-terminal regions (Additional file 2: Figure S2a). In *U. maydis*, a glycine-rich domain of 37 aa at the C-terminus was deleted without affecting biological activity, however, cysteine residues are necessary for secretion of Pep1 [26]. The molecular function of these two regions in *S. scitamineum* may be similar to that in *U. maydis*. Interestingly, phylogenetic tree based on Pep1 (Additional file 2: Figure S2b) is in accordance with that based on the whole genome (Figure 2), implying that Pep1 can probably be used as a molecular marker for phylogenetic analysis or even for race identification in smut fungi.

### Secondary metabolic pathways

Plant pathogenic fungi produce diverse secondary metabolites that aid in pathogenicity, such as host selective toxins. We identified 145 putative secondary metabolite genes and 11 gene clusters in the *S. scitamineum* genome. There are 20, 26 and 18 genes predicted to encode polyketide synthases (PKS), non-ribosomal peptide synthetases (NRPS), and terpenes, respectively. Two NRPS gene clusters may participate in iron-chelating siderophores ferrichrome and ferrichrome A biosynthesis. One terpene gene cluster may involve the conversion of gibberellins.

**Figure 10:**
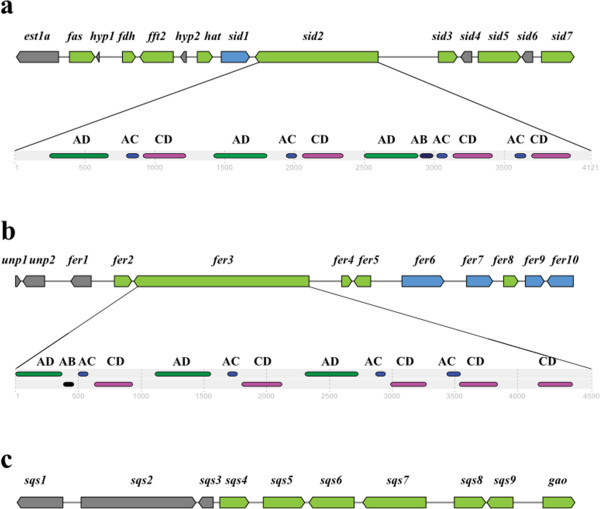
**Three gene clusters in the genome of** ***Sporisorium scitamineum*** **. (a)** Physical map of ferrichrome gene cluster (SmutADNA4_GLEAN_10002720-10002733). Coding regions are indicated with arrows and are putatively involved in ferrichrome biosynthesis (green yellow), siderophore transport (blue), or unknown functions (grey). Location of InterPro domains in NRPS SmutADNA4_GLEAN_10002728 is as bottom. AD, AMP-dependent synthetase/ligase domain (green), IPR00873; AC, acyl carrier domain (purple), IPR009081; CD, condensation domain (red), IPR001242; AB, AMP-binding enzyme C-terminal domain (black), IPR025110. **(b)** Physical map of ferrichrome A gene cluster (SmutADNA4_GLEAN_10004009-10004020). Coding regions are indicated with arrows and are putatively involved in ferrichrome A biosynthesis (green yellow), siderophore transport (blue), or unknown functions (grey). Location of InterPro domains in NRPS SmutADNA4_GLEAN_10004013 is as bottom. AD, AMP-dependent synthetase/ligase domain (green), IPR00873; AC, acyl carrier domain (purple), IPR009081;CD, condensation domain (red), IPR001242; AB, AMP-binding enzyme C-terminal domain (black), IPR025110. **(c)** Physical map of terpene gene cluster (SmutADNA4_GLEAN_10000695-10000704). Coding regions are indicated with arrows and are putatively involved in terpene biosynthesis (green yellow) or unknown functions (grey).

Iron is an important element for many essential processes in living organisms. To acquire iron, *S. scitamineum* uses two iron-chelating siderophores to uptake iron from host sugarcane cells. In Figure 10a, one NRPS gene cluster including 14 genes, is involved in ferrichrome generation. The core gene SmutADNA4_GLEAN_10002728 encodes ferrichrome siderophore peptide synthetase, and encodes 4,121 amino acids with 76% identity to the NRPS Sid2 of *U. maydis* 521 [28]. The gene has three AMP-dependent synthetase/ligase domains, four acyl carrier domains (IPR009081) to activate the cognate amino acid, four condensation domains to catalyze peptide bond formation between the amino acyl and peptidyl substrates of adjacent domains and one AMP-binding enzyme C-terminal domain [29]. The other NRPS gene cluster consists of 12 genes, responsible for ferrichrome A biosynthesis (Figure 10b). The skeleton gene SmutADNA4_GLEAN_10004013 is located in the fifth gene cluster and encodes siderophore peptide synthetase. It encodes 4,590 amino acids with 75% identity to the NRPS Fer3 (CBQ70842.1) of *S. reilianum* SRZ2, which is composed of three AMP-dependent synthetase/ligase domains, five acyl carrier domains, four condensation domains and one AMP-binding enzyme C-terminal domain.

Two NRPSs involved in siderophore biosynthesis have also been identified in the ascomycete *Cochliobolus heterostrophus* and the ferricrocin biosynthesis enzyme is conserved in other ascomycetes [30, 31]. *A. fumigatus* is one of the most common *Aspergillus* species to cause disease in individuals with an immunodeficiency *A. fumigatus* produces four siderophores, fusarinine C, triacetylfusarinine C, ferricrocin and hydroxyferricrocin [32]. *A. fumigatus* has been recognized as a model species to study fungal siderophore biosynthetic pathways. These two NRPS gene clusters require further study to describe the function of gene members in this cluster by metabolite detection methods such as High Performance Liquid Chromatography (HPLC), Gas Chromatography (GC) and mass spectrometry. Molecular biology experiments also need to be conducted such as knock-out, knock-in, and heterogeneous expression.

In Figure 10c, the terpenes gene cluster is composed of three unknown function genes and seven biosynthesis related genes. The gene SmutADNA4_GLEAN_10000704, which encodes gibberellin 20-oxidase, is known to encode 400 amino acids that have 90% identity with sr15265 (CBQ69196.1) of *S. reilianum* SRZ2. Gibberellin 20-oxidase is a key oxidase in the biosynthesis of gibberellin (GA). Gibberellin 20-oxidase catalyzes the conversions of GA12 and GA53 to GA9 and GA20 respectively, via a three-step oxidation at C-20 of the GA skeleton. GA35 probably plays an important role in *S. reilianum* resistance in infected *Sorghum bicolor*, however, GA20 has no effect [33]. This implies that the gene cluster is involved in weakening the resistance of sugarcane to smut disease and contributes to the successful infection of *S. scitamineum*.

With GC-MS detection, *S. scitamineum* can biosynthesize 65 compounds and secrete 13 extracellularly (Additional file 1: Table S9). The intracellular metabolites are mainly esters and the distribution of molecular weight (MW) is from 99 to 475 amu. However, the extracellular metabolites are alcohol (2), aldoketones (4), alkanes (2), and carboxylic acids (2) and no esters. The extracellular metabolites exhibited a smaller MW distribution (96-264 amu). Neither intracellular nor extracellular metabolites had strong infection to sugarcane in the inoculation experiments by supernatant (cell-free extracts) and precipitation of fungal elicitor. The questions of whether this was due to the application of mixed metabolites, and if only the single or some secreted metabolites function(s) in the infection process, require further investigation through additional inoculation validation. Determining the function of each metabolite in this process could elucidate the corresponding mechanism causing sugarcane smut.

Cytochrome P450s play an important role in various hydroxylation and oxidation processes including those associated with secondary metabolites as well as the breakdown of toxins and other xenobiotic compounds [34]. Plant pathogenic fungi use a wide range of strategies to gain access to the carbon sources and nitrogen sources of their host plants and to counter the plant defense response. Transporters are involved in toxin and effector secretion as well as nutrition uptake. There are 18 P450s and 129 transporter genes (Table 5) in the genome of *S. scitamineum*. A large proportion of transporters belong to the major facilitator superfamily (MFS) (74), but the ABC superfamily (17), P-type ATPase (P-ATPase) Superfamily (18), and Sugar/inositol transporter (20) are all well represented. These four smut fungi have similar composition of P450s and transporters. Compared to the typical pathogens *M. grisea* or *Fusarium graminearum* [35], the MFS number of *S. scitamineum* is small. It is unsurprising that few metabolites could be secreted extracellularly.

**Table 5:** **Comparative analysis of P450s and transporter genes among various plant pathogen fungi**

Protein families	Biotroph				Obligate_biotroph		Hemibiotroph		Necrotroph			
	***Ss***	***Uh***	***Um***	***Sr***	***Mlp***	***Pgt***	***Mo***	***Fg***	***Vd***	***Fo***	***Fv***	***Nh***
Cytochrome P450	18	25	31	25	33	23	134	126	72	238	148	173
Major facilitator superfamily	74	70	74	74	65	57	181	245	190	179	236	447
ATP-binding cassette (ABC) superfamily	17	14	19	16	9	12	27	32	18	27	32	38
Sugar/inositol transporter	20	18	18	21	19	13	52	87	66	52	115	145
P-type ATPase (P-ATPase) superfamily	18	8	17	18	17	16	12	27	22	23	31	34

*Ss*, *Sporisorium scitamineum*; *Uh*, *Ustilago hordei*; *Um*, *Ustilago maydis*; *Sr*, *Sporisorium reilianum*; *Mlp*, *Melampsora laricis-populina*;

*Pgt*, *Puccinia graminis* f. sp. *tritici*; *Mo*, *Magnaporthe oryzae*; *Fg*, *Fusarium graminearum*; *Vd*, *Verticillium dahliae*; *Fo*, *Fusarium oxysporum*;

*Fv*, *Fusarium verticillioides*; *Nh*, *Nectria haematococca*.

## Conclusions

Although sugarcane smut was first reported in 1877, little is known about its pathogenic mechanisms. The *S. scitamineum* genome sequence and our comparative analysis with the genome sequences of *U. maydis*, *S. reilianum* and *U. hordei* have shed new light on the pathogenic mechanisms of this fungus and have provided insights into aspects of genome evolution, biotrophy features, carbohydrate degrading enzymes and secondary metabolic pathways likely to be common to all smut fungi. These results represent the initial step in realizing the full potential of these genomes. As a result of the genome analysis, efforts are underway to validate the function of all these potential pathogenicity determinants, especially the virulence associated genes. However, the genome sequence of *S. scitamineum* provides only a first glimpse into the genomic basis of pathogenic mechanisms of sugarcane smut. These efforts and ongoing sequencing projects for additional sugarcane fungi will provide extraordinary opportunities for comparative analyses that will expand our understanding of the interaction between sugarcane and fungi in this agriculturally and industrially important crop. Further sequencing of *S. scitamineum* isolates from different geographical locations or different host varieties will lead to comprehensive population genetic analysis.

## Methods

### Ethics statement

No specific permissions are required for these locations/activities. The field studies do not involve endangered or protected species and are conducted in accordance with local legislation.

### Strain, growth condition, and genomic DNA and RNA isolation

Smut infected whips were collected from sugarcane cultivar “ROC”22 maintained at the Key Laboratory of Sugarcane Biology and Genetic Breeding, Ministry of Agriculture (Fuzhou, China). Teliospores were mixed, sealed in plastic bags and then stored at 4°C. These samples were subjected to gradient dilution with sterile water and then plated onto potato dextrose agar (PDA) medium which contained 100 μg/mL streptomycin (Sangon Biological Engineering Technology & Services Co., Ltd, Shanghai, China). Plates were incubated in the dark at 28°C for 5 d. Single colonies were transferred onto the new PDA medium and cultured at 28°C for 7 d. A single colony named SmutA (haploid) was transferred into potato dextrose water (PDW) medium with 100 μg/mL streptomycin and incubated at 28°C, 200 rpm for 5 d. DNA from fungal hyphae was extracted using the SDS method and eluted with sterile water containing 100 μg/mL RNase A. The DNA sample was quantified by the NanoVue Plus Spectrophotometer (GE Healthcare, NJ, USA) using the absorbance at 260 nm and 280 nm. The DNA was submitted to genome sequencing at BGI-Shenzhen (Shenzhen, China). For fungal elicitor preparation, the SmutA was also transferred into potato dextrose water (PDW) medium with 100 μg/mL streptomycin and incubated at 28°C, 200 rpm for 15 d.

A smut spore suspension containing 5 × 10^6^ spores/mL in 0.01% (v/v) Tween-20 was needle-inoculated on to buds of sugarcane cultivar “ROC”22 to create the treatment group. All the inoculated buds were then cultured in an incubator at 28°C ±0.5°C in the condition of 12 h light/12 h dark. Five phenotypically normal buds were then collected at each of the time points of 0, 12, 24, 48, 96 and 120 h. Samples were immediately frozen in liquid nitrogen and stored at –80°C. Total RNA of these inoculated buds was isolated using the TRIzol® reagent according to the manufacturer’s instructions (Life Technologies Co. Ltd., CA, USA). Dried RNA samples were dissolved in diethylpyrocarbonate-treated water. RNA quality was assessed on 1.0% denaturing agarose gels, and the quantity was verified using the NanoVue Plus Spectrophotometer (GE Healthcare, NJ, USA) and the Agilent 2100 Bioanalyzer (Agilent Technologies Co. Ltd., CA, USA). The total RNA of “ROC”22 after pathogen inoculation for 0, 12, 24, 48, 96 and 120 h were subjected to an analysis of expression pattern of each gene by Real-time quantitative PCR (RT-qPCR).

### Genome sequencing and assembly

From the genomic DNA of *S. scitamineum*, 170 bp and 500 bp PCR-free as well as 6 kb and 10 kb DNA sequencing libraries were constructed. A total of 6,316 Mb was generated by the IlluminaHiseq™ 2000 at BGI-Shenzhen (Shenzhen, China). To ensure the accuracy of assembly, reads with 20 low-quality (≤Q2) bases, 5.5% Ns, or 15 bp overlap between adapter and duplications were filtered. The short reads from the four libraries were assembled by *SOAPdenovo* 1.04 [36] with optimal assembly acquired with the key parameter K = 55.

### Comparative genome analysis

Gene clustering was conducted with OrthoMCL [37] by setting the main inflation value as 1.5 and other parameters at default setting. Gene clustering was done for four smut fungi including *S. scitamineum*, *U. maydis*, *U. hordei* and *S. reilianum* for gene family analysis. After clustering, we generated multiple alignments of protein sequences for each gene family using MUSCLE [34] and converted the protein alignments to CDS alignments.

The single-copy core genes of those four fungi and the outgroup species (*Aspergillus nidulans*) were identified using OrthoMCL [37]. After the core-gene identification, we constructed the multiple alignments with MUSCLE [38]. The subprogram phyml of TreeBeST (http://treesoft.sourceforge.net/treebest.shtml) was used to construct a phylogenetic tree with default parameters and 1,000 bootstrap samples.

### Gene prediction and annotation

To predict genes in the assembled genome, both homology-based and *de novo* methods were used. For the homology-based prediction, proteins of *U. maydis* strain 521 were mapped onto the assembled genome using Genewise [39]. For *de novo* prediction, Augustus software program [40] was employed using appropriate parameters. Data from these complementary analyses were merged to produce a non-redundant reference gene set using GLEAN (http://glean-gene.sourceforge.net/).

Repeat sequences were identified by Repeat Masker version 3.3.0 with Repbase version 18.05, and the following parameters: -–nolow –no_is –norna -s –engine wublast –parallel 1 –lib lib; Repeat Protein Mask was also used with parameters: -noLowSimple, -pvalue = 1e-4 [41]. Non-coding RNA was predicted by rRNAmmer 1.2, tRNAscan-SE 1.23, and Rfam 10.1.

The protein-encoding genes were annotated through BLASTp searches in the SwissProt (2012-06), GO (release: 1.419), COG (release: 20090331), KEGG (release: 59), and NR (2013-09-04) databases, at the threshold of e-value ≤ 1 × e^-5^. The best hit was filtered using a 50% identity cut-off value.

To identify proteins involved in carbohydrate metabolism, we used the Carbohydrate Active Enzymes (CAZy) database (http://www.cazy.org/). Pathogenicity and virulence associated genes were identified using the PHI-base database (http://www.phibase.org/). The PHI-base database catalogs experimentally verified pathogenicity, virulence, and effector genes from fungal, oomycete, and bacterial pathogens which infect animal, plant, fungal and insect hosts. Putative secondary metabolites (PKS and NRPS) were identified by using antiSMASH [42]. Cytochrome P450s were identified by the Cytochrome P450 Database (http://drnelson.uthsc.edu/CytochromeP450.html).

### Effector protein

The secreted proteins (effectors) of *S. scitamineum* were analyzed using several prediction algorithms. TargetP 1.1 (http://www.cbs.dtu.dk/services/TargetP/) was used to predict the cleavage sites of the predicted presequences with the “Perform cleavage site predictions” option. SignalP 4.0 (http://www.cbs.dtu.dk/services/SignalP-4.0/) was used to perform signal peptide cleavage site prediction. Transmembrane helices in the proteins were predicted using TMHMM 2.0 (http://www.cbs.dtu.dk/services/TMHMM-2.0/). The proteins that contained signal peptide cleavage sites and no transmembrane helices were selected as effectors. The proteins with lengths less than 200 amino acids or Cysteine content less than 1.5% were removed and the candidate effectors were obtained. CSEP clustering was conducted with OrthoMCL [37] by setting the main inflation value at 1.5 and other parameters at default values. GPCRs sequences were evaluated for seven transmembrane regions by Phobius [43] and TMHMM 2.0 with default settings.

### Fungal elicitor preparation and GC-MS detection

The fungal elicitor includes two parts, that from supernatant (cell-free extracts) and the part from precipitation. For the elicitor preparation from cell-free extracts, 100 ml of the 15 d culturing liquid medium containing SmutA was filtered with four pieces of gauze, centrifuged at 3,000 rpm for 20 min, filtered with a 0.22 μm filter (Merck-Millipore, Tullagreen, Carrigtwohill, Co. Cork, IRL), and kept at 4°C until use [44]. For the elicitor preparation from the precipitation, 100 ml of the 15 d culturing liquid medium containing SmutA was centrifuged at 5,000 *g* for 10 min. Mycelia were then harvested, washed in distilled water, dried with filter paper, weighted, and then ground to a fine powder in liquid nitrogen. The powder was suspended with 25 ml of 10 mM Tris-HCl (pH 8.8). Following 5,000 *g* centrifugation for 10 min at 4°C, 20 ml of 80% (v/v) methanol was used to suspend the pellet, and the mixture was shaken for 4 h at 38°C. After a further centrifugation at 5,000 *g* for 20 min at 4°C, the pellet from the mixture was suspended once with 5 ml methanol and dried under nitrogen air flow. The dried pellet was washed with 10 ml of 10 mM phosphate buffer (pH 6.8) again, re-suspended with 25 ml of the same buffer and the mixture was autoclaved for 20 min at 120°C. Finally, the mixture was centrifuged at 10,000 *g* for 20 min at 4°C and the clear supernatant was collected and kept at 4°C until use [45].

For detection analysis, the cell-free extracts and the extracts from the precipitation were dried at -80°C with a freeze vacuum drier and then dissolved with 10 ml HPLC/spectro-methanol. These samples were analyzed by a GC-MS analyzer (Agilent 7890A-5975C). The column was a DB-5ms elastic quartz capillary column (30 m × 250 μm × 0.25 μm). The carrier gas was helium at a flow rate of 1 ml/min and the injection volume was 1μl. The column temperature program was 50°C for 2 min, which ramped from 50°C to 250°C at 6°C /min, and held at 250°C for 150 min. In the mass spectroscopy, the ion source temperature was 230°C, and the ionization mode was EI (70 eV). The constituents were identified and compared with the NIST08 standard MS database.

### Carbon and nitrogen source utilization

For determining the growth profiles on different carbon and nitrogen substrate, we modified the Czapek for use as basal medium by supplementing 3.0% agar with 0.2% NaNO_3_, 0.1% KH_2_PO_4_, 0.05% MgSO_4_•7H_2_O, 0.05% KCl, 0.01% FeSO_4_•7H_2_O and 3.0% sucrose [46]. The original pH was adjusted to 6.5 unless otherwise indicated. All the chemicals or reagents were purchased from China National Medicines Corporation Ltd. (Shanghai, China). All the chemicals used in the present study were AR grade or equivalent in purity. Millipore water was used for preparation of all media. For strain growth, fungus cake with Φ = 5 mm was inoculated onto the medium flat plate and the culture was incubated at 28°C in a thermostat incubator for seven days, and then photographed. PDA was set as the internal control medium. Three replicates were conducted.

In carbon utilization experiments, the basal medium was supplemented with 90 mM of the carbon source that had equivalent carbon content to that of sucrose. Eight types of carbon substrates, including glucose, maltose, fructose, lactose, soluble starch, D-mannitol, D-mannose, and D-galactose were added separately at a final concentration of 5.0 mg/mL. In nitrogen utilization studies, the basal medium was adjusted with 24 mM of the nitrogen source that had equivalent nitrogen content to that of sodium nitrate. Twenty-two kinds of nitrogen substrate, including ammonium sulphate, ammonium acetate, leucine, tryptophan, isoleucine, threonine, cysteine, phenylalanine, histidine, aspartate, asparagine, glutamine, arginine, glutamate, glycine, lysine, serine, valine, alanine, proline, tyrosine, and ethionine were added separately in the medium at a final concentration of 10.0 μM.

### Gene expression

The method of RT-qPCR followed the instructions of the SYBR Green Master (ROX) (Roche, China) on a 7500 Real-time PCR system (Applied Biosystems, USA). The *GAPDH* gene (Forward primer: CACGGCCACTGGAAGCA; Reverse primer: TCCTCAGGGTTCCTGATGCC) was chosen as the internal control of the RT-qPCR. According to the sequence of each target gene of SmutA, a pair of specific primers was designed using the Primer Premier 5.0 software [47]. RT-qPCR was carried out with the FastStart Universal SYBR Green Master (ROX) in a 20 μL volume containing 10.0 μL FastStart Universal SYBR Green PCR Master (ROX), 0.5 μM of each primer, and a 1.0 μL template (5 × diluted cDNA). RT-qPCR with distilled water as the template was performed as a negative control. The RT-qPCR reaction conditions were held at 50°C for 2 min, 95°C for 10 min, 40 cycles of 95°C for 15 s, and 60°C for 1 min [47]. When the reaction was complete, the melting curve was analyzed. Each RT-qPCR was repeated three times. The 2^-△△Ct^ method was adopted to analyze the RT-qPCR results [47, 48]. The primer information of candidate virulent factors is listed in the Additional file 1: Table S10.

### Data access

All data contributing to this genome initiative has been deposited at the NCBI under BioProject PRJNA240344; the genome accession number is [JFOL00000000]. The genome version described in this paper is the first version and the accession number is [JFOL01000000].

## Electronic supplementary material

Additional file 1: Table S1: Data used for *S. scitamineum* genome assembly and scaffolding. **Table S2.** Gene prediction in the *S. scitamineum* genome. **Table S3.** Protein database annotation result for proteins of *S. scitamineum*. **Table S4.** Noncoding genes in the *S. scitamineum* genome. **Table S5.** Repeat elements in the *S. scitamineum* genome. **Table S6.** Expression of candidate genes for encoding pant cell wall degradation enzymes of *S. scitamineum* at six stages in the infection process. **Table S7.** Candidate virulence associated genes of *S. scitamineum* compared against pathogen-host interaction (PHI) database and their expression at six stages in the infection process. **Table S8.** Candidate genes for Secreted Effector Proteins in the *S. scitamineum* genome (aa length > 200 aa & Cys (%) > 1.5%) and their expression at six stages in the infection process. **Table S9.** Sixty-five compounds in the elicitor of *S. scitamineum* detected by GC-MS. **Table S10.** Primer information of Real-time quantitative PCR for candidate virulent factors. (XLSX 72 KB)

Additional file 2: Figure S1: The K-mer distribution and GC-depth analysis. **(a)** 15-mer depth distribution of 32 X data. Analysis X-coordinate is depth, and Y-coordinate is frequency. **(b)** GC content and depth correlative analysis. X-coordinate is GC content, and Y-coordinate is average depth. Through calculating GC content and average depth, we can analyze whether GC bias exists. If not seriously biased, this scatter diagram takes on the shape similar to Poisson distribution, that is there will be a peak near the GC content of the genome, and the more deviation from it, the lower the depth is. **Figure S2.** Pep1 is conserved among *Sporisorium scitamineum* and other sequenced smut fungi. **(a)** Sequence alignment of *S. scitamineum* Pep1 (Ss), *S reilianum* Pep1 (Sr), *U. maydis* Pep1 (Um), and *U. hordei* Pep1 (Uh). Identical amino acids are highlighted in green. Red boxes: conserved cysteine residues; black box: putative N-terminal secretion signal; blue box: poorly conserved glycine-rich C-terminal region. **(b)** Phylogenetic tree based on Pep1 multiple sequence alignment. (DOCX 844 KB)
