# Erratum: Genome sequencing of *Sporisorium scitamineum* provides insights into the pathogenic mechanisms of sugarcane smut

**DOI:** 10.1186/s12864-015-1336-4

**Published:** 2015-03-26

**Authors:** Youxiong Que, Liping Xu, Qibin Wu, Yongfeng Liu, Hui Ling, Yanhong Liu, Yuye Zhang, Jinlong Guo, Yachun Su, Jiebo Chen, Shanshan Wang, Chengguang Zhang

**Affiliations:** Key Laboratory of Sugarcane Biology and Genetic Breeding, Ministry of Agriculture, Fujian Agriculture and Forestry University, Fuzhou, 350002 China; BGI-Shenzhen, Main Building 11/F, Beishan Industrial Zone, Shenzhen, 518083 Yantian District China; College of Life Science, Fujian Agriculture and Forestry University, Fuzhou, 350002 China

## Erratum to: BMC Genomics 2014, 15 :1184 doi:10.1186/1471-2164-15-1184

Following publication of this article it came to our attention that we neglected to acknowledge the inspiration for figure four (Figure 1 here) and text associated with the figure provided by Guus Bakkeren and colleagues http://dx.doi.org/10.1016/j.fgb.2008.04.005 (9). We have included text to replace that appropriated from the review article by Bakkeren and colleagues. We sincerely apologise for the oversight.

## Results and discussion

### Characterization of mating type loci in *S. scitamineum*

For mating to occur, two haploid cells of different mating-type need to recognize each other and fuse to form the infectious dikaryon. Mating is regulated by two loci, *a* and *b*, which harbor conserved genes. At the *a* locus, these genes encode pheromones and pheromone receptors while at the *b* locus two subunits of a heterodimeric transcription factor are encoded [1].

The bipolar species *S. scitamineum* and *U. hordei* as well as the tetrapolar species *U. maydis* and *S. reilianum* possess one divergently transcribed gene pair that encode the homeodomain proteins bE (HD1) and bW (HD2). The MAT-1 locus, gene order, orientation, as well as the genomic context are conserved in the *b* mating-type genes except for the *U. hordei* MAT-2 locus figure four (Figure 1 here). Interestingly, both *bE* and *bW* mating-type genes are present in the genomes of Ustilaginaceae including the two genera of *Ustilago* and *Sporisorium*.

In addition to the *b* mating-type complexes, smut fungi contain genes necessary for cell–cell recognition which are located in the *a* mating type loci. The detailed structure of these loci has been determined for both the MAT-1 alleles of *S. scitamineum* and *U. hordei*, an allele of *U. maydis,* and for all three alleles of *S. reilianum* figure four (Figure 1 here). Both *U. maydis* and *U. hordei* have two alleles of an *a* mating system with one pheromone receptor (*pra*) and one functional pheromone gene (*mfa*) per locus.

In *S. scitamineum* and *U. hordei* which have a bipolar mating system, the *a* an*d b* loci are linked and the mating-type locus (MAT) segregates as one locus. However, in tetrapolar species such as *S. reilianum* and *U. maydis*, these genetic loci segregate independently [2]. In *S. scitamineum*, the *a* locus encodes a lipopeptide with pheromone and pheromone membrane receptor functions responsible for cell recognition and compatible hyphal fusion**,** whereas the *b* locus encodes transcription factors that control the expression of genes responsible for the maintenance of the dikaryotic hyphal growth in plants figure four (Figure 1 here). During their life cycle, *S. scitamineum* presents two distinct monokaryotic and dikaryotic stages. The monokaryotic stage is marked by haploid cells that grow saprophytically and are not able to cause disease, while in the second phase, dikaryotic hyphae are formed by mating (sexual crossing) and are able to infect the host. The induction of the pathogenic program in *S. scitamineum* implies not only strong morphological changes (from yeast-like to hyphal) but also genetic changes (haploid to dikaryotic transition).

Evolution of bipolar mating in *S. scitamineum* may have been beneficial for the fungus because it promoted inbreeding and stabilization of the genome. The same process has been proved to be beneficial for the transposon elements (TEs). A study concluded that inbreeding helped fix TEs within a population in *U. hordei* [10]. In tetrapolar species, such as *U. maydis* and *S. reilianum*, outcrossing increases heterozygosity [2].

Overall, sequence analysis and comparison of the mating-type regions of tetrapolar and bipolar smut fungi revealed that they are not fundamentally different. Bipolar and tetrapolar smuts as well as related species contain the genes for these *a* and *b* mating-type complexes. In the bipolar species *S. scitamineum* and *U. hordei*, these mating-type complexes are encoded on the same chromosome and in a recombination-suppressed region ensuring genetic linkage.

**Figure 1:**
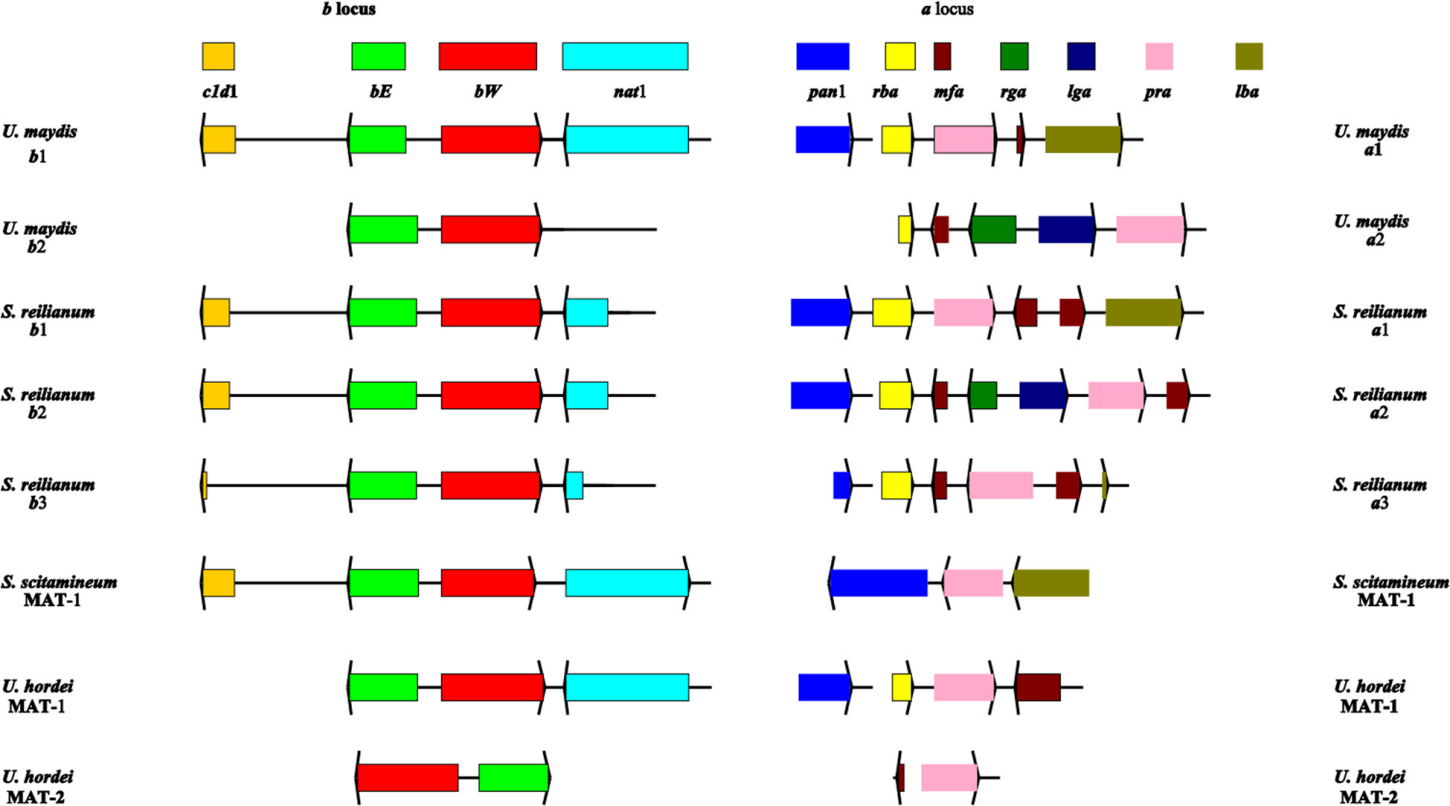
**Genetic organization of the mating-type loci of four smut fungi.** Genes are indicated by arrows with the arrow denoting the direction of transcription. Related genes are denoted by the same color and respective gene functions are explained in the lower part of the Figure. *indicates that the relative order and orientation of these genes have not been determined. In the tetrapolar species, *U. maydis and S.reilianum,* the a and b specific sequences reside on different chromosomes, while they are linked by spacer regions (which are not drawn to scale and whose length is indicated) in the bipolar species *U. hordei* and *S. scitamineum*. The black bars on top of the figure indicate the regions of the b locus, which covers the two homeodomain protein genes bE and bW, and the a locus (that expands to different length in the different loci, indicated by a broken line) from the lba gene to the rba gene. Sequence information was obtained from the following Accession Numbers: AF043940, AM118080, AF184070, AF184069, Z18531, AJ884588, AJ884583, AJ884590, AJ884585, AJ884589, AJ884584, U37796, M84182, AACP01000083 and AACP01000013. Refer to Bakkeren and colleagues in *Fungal Genet Biol* [1], we have added the species *S. sporisorium,* and deleted M. globosa and *C. neoformans* to obtain the genetic organization of the mating-type loci of four smut fungi as shown in figure four (Figure 1 here).
