# Supplementary material for: Genome sequencing of Sporisorium scitamineum provides insights into the pathogenic mechanisms of sugarcane smut
Source: BMC Genomics. 2014 Nov 19;15(1):996. doi: 10.1186/1471-2164-15-996 (PMC4246466; doi:10.1186/1471-2164-15-996)
Supplement: Supplementary file 2 — Additional file 2: Figure S1: The K-mer distribution and GC-depth analysis. (a) 15-mer depth distribution of 32 X data. Analysis X-coordinate is depth, and Y-coordinate is frequency. (b) GC content and depth correlative analysis. X-coordinate is GC content, and Y-coordinate is average depth. Through calculating GC content and average depth, we can analyze whether GC bias exists. If not seriously biased, this scatter diagram takes on the shape similar to Poisson distribution, that is there will be a peak near the GC content of the genome, and the more deviation from it, the lower the depth is. Figure S2. Pep1 is conserved among Sporisorium scitamineum and other sequenced smut fungi. (a) Sequence alignment of S. scitamineum Pep1 (Ss), S reilianum Pep1 (Sr), U. maydis Pep1 (Um), and U. hordei Pep1 (Uh). Identical amino acids are highlighted in green. Red boxes: conserved cysteine residues; black box: putative N-terminal secretion signal; blue box: poorly conserved glycine-rich C-terminal region. (b) Phylogenetic tree based on Pep1 multiple sequence alignment. (DOCX 844 KB) [file 12864_2014_6669_MOESM2_ESM.docx]

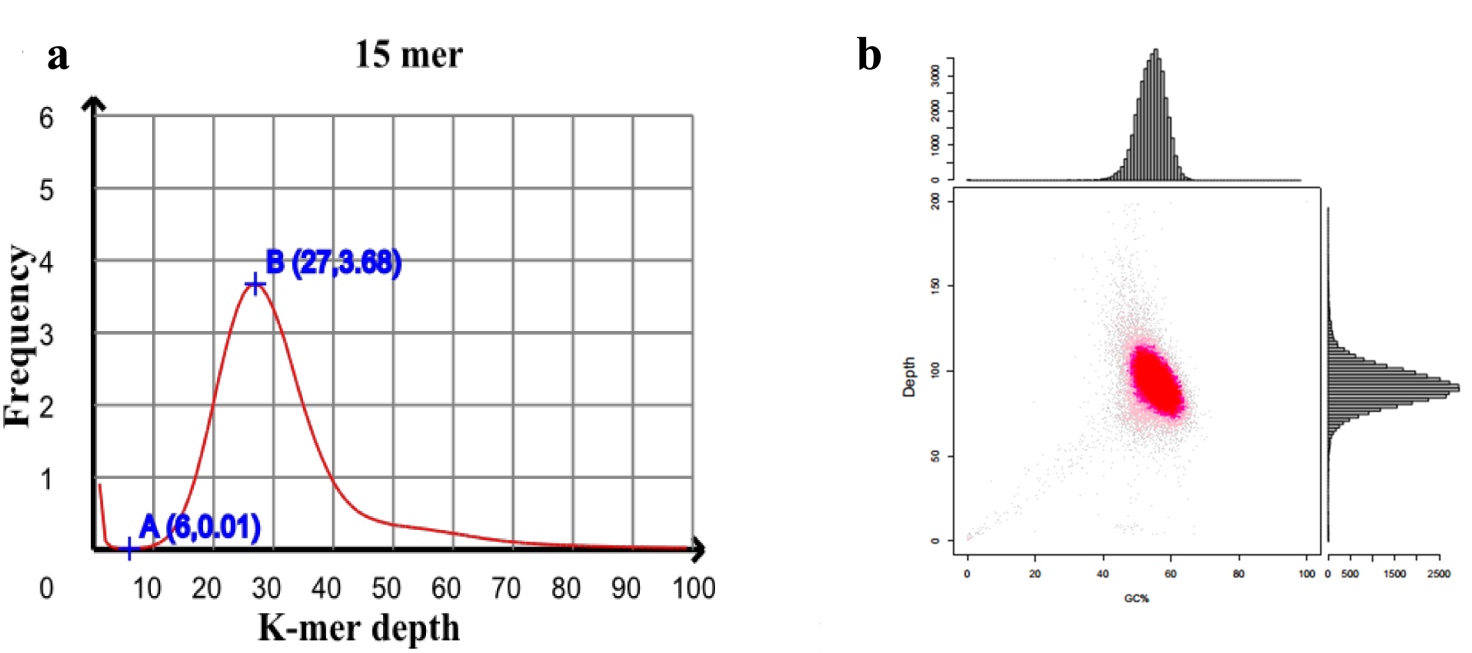


**Figure S1 The K-mer distribution and GC-depth analysis.**


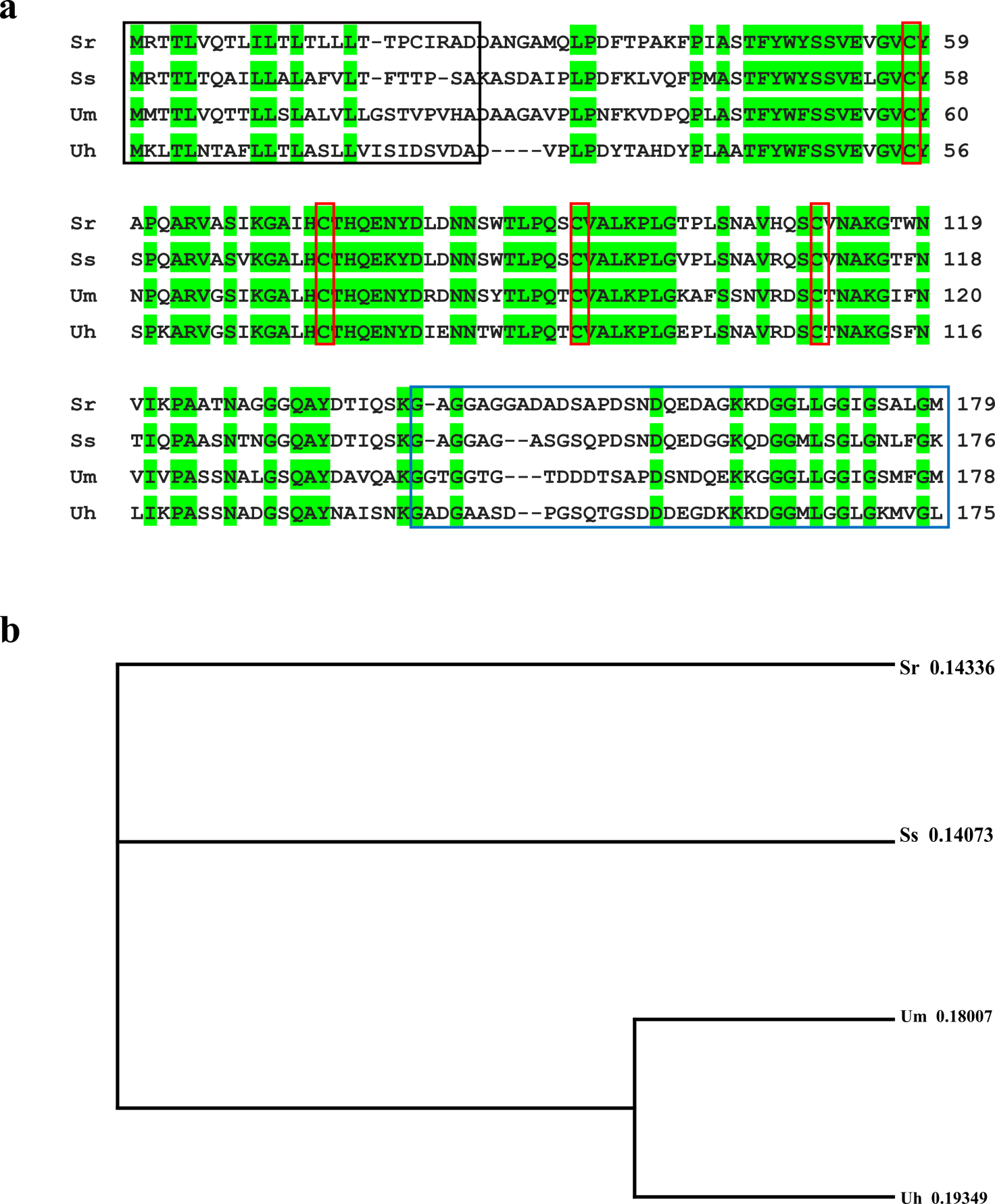


**Figure S2 Pep1 is conserved among *Sporisorium scitamineum* and other sequenced smut fungi.**
